# Supplementary material for: Artificial cysteine-lipases with high activity and altered catalytic mechanism created by laboratory evolution
Source: Nat Commun. 2019 Jul 19;10:3198. doi: 10.1038/s41467-019-11155-3 (PMC6642262; doi:10.1038/s41467-019-11155-3)
Supplement: Supplementary file 1 — Supplementary Information [file 41467_2019_11155_MOESM1_ESM.pdf]

Supplementary Information

Artificial Cysteine-Lipases with High Activity and Altered Catalytic Mechanism Created by Laboratory Evolution

Yixin Cen[1,2], Warispreet Singh[3,4], Mamatjan Arkin[1], Thomas S. Moody[4], Meilan Huang[3,*], Jiahai Zhou[2,*], Qi Wu[1,*] & Manfred T. Reetz[5,6,*]

[1]Department of Chemistry, Zhejiang University, Hangzhou, 310027, P. R. China;

[2]State Key Laboratory of Bio-organic and Natural Products Chemistry, Shanghai Institute of Organic Chemistry, Chinese Academy of Sciences, Shanghai, 200032, China;

[3]School of Chemistry and Chemical Engineering, Queen's University, David Keir Building, Stranmillis Road, Belfast BT9 5AG, Northern Ireland, UK;

[4]Almac Sciences, Department of Biocatalysis and Isotope Chemistry, Almac House, 20 Seagoe Industrial Estate, Craigavon BT63 5QD, Northern Ireland, UK;

[5]Max-Planck-Institut für Kohlenforschung, Kaiser-Wilhelm-Platz 1, 45470, Mülheim an der Ruhr, Germany;

[6]Chemistry Department, Philipps-University, Hans-Meerwein-Str. 4, 35032 Marburg, Germany.

* Corresponding authors.

M.H.: m.huang@qub.ac.uk

J.Z.: jiahai@mail.sioc.ac.cn

Q.W.: wuqi1000@163.com

M.T.R.: reetz@mpi-muelheim.mpg.de

Table of Contents

**Supplementary Figures 1-20**	**2**
**Supplementary Tables 1-5**	**22**
**Supplementary Methods.**	**27**
**Supplementary References**	**32**

Supplementary Figures

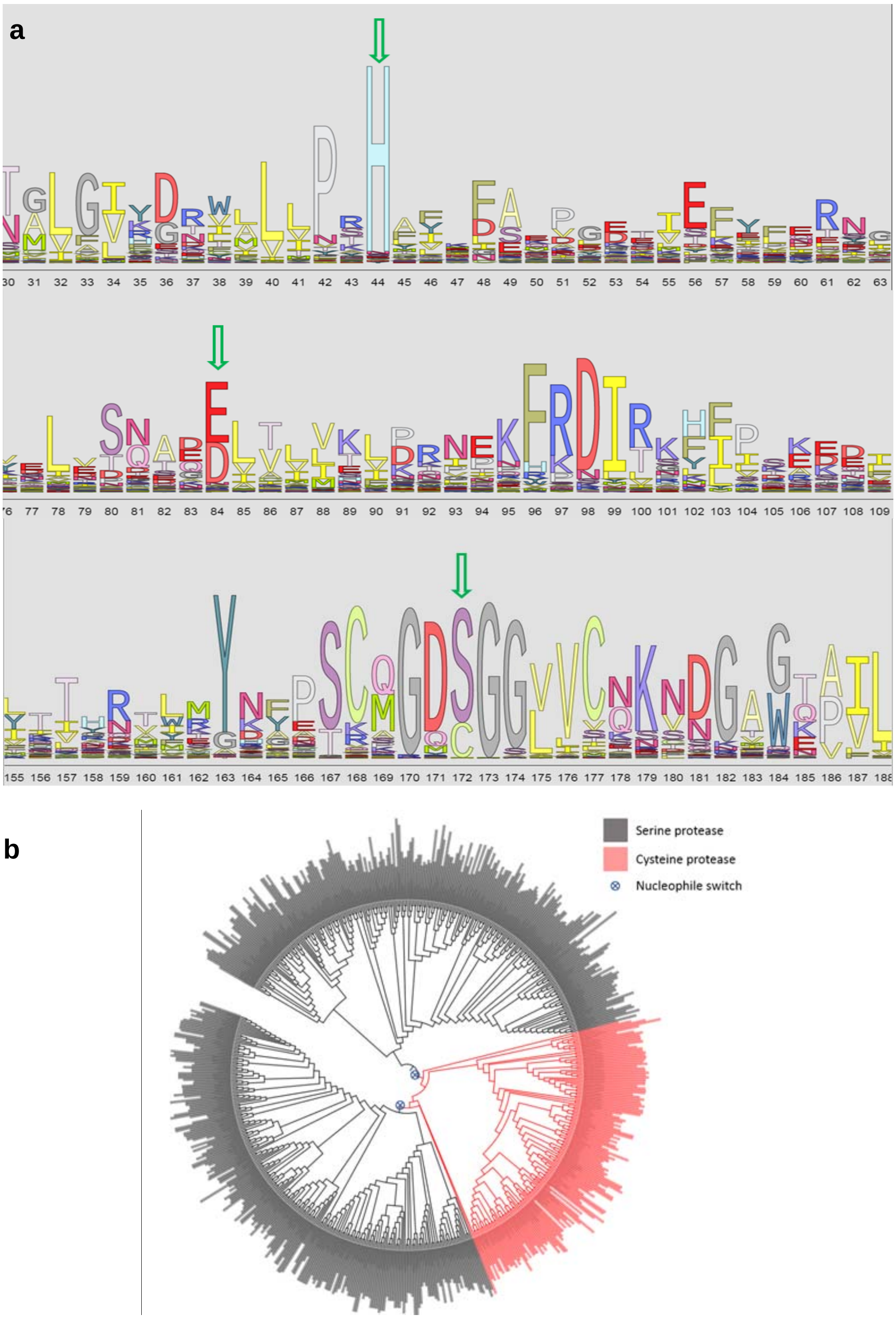

Supplementary Figure 1. Multiple sequence alignment and phylogenetic analysis of proteases. **a,** Alignment of 1000 sequences of serine proteases and cysteine proteinase. The reference structure is Hepatitis A virus 3c proteinase (triad: C172-H44-D84) (PDB: 1HAV). The conserved catalytic triad C/S-H-D/E are labelled using blue arrows. **b,** Maximun-likelihood phylogenetic tree was estimated using the WAG model based on 644 sequences of serine proteases and cysteine proteinase.

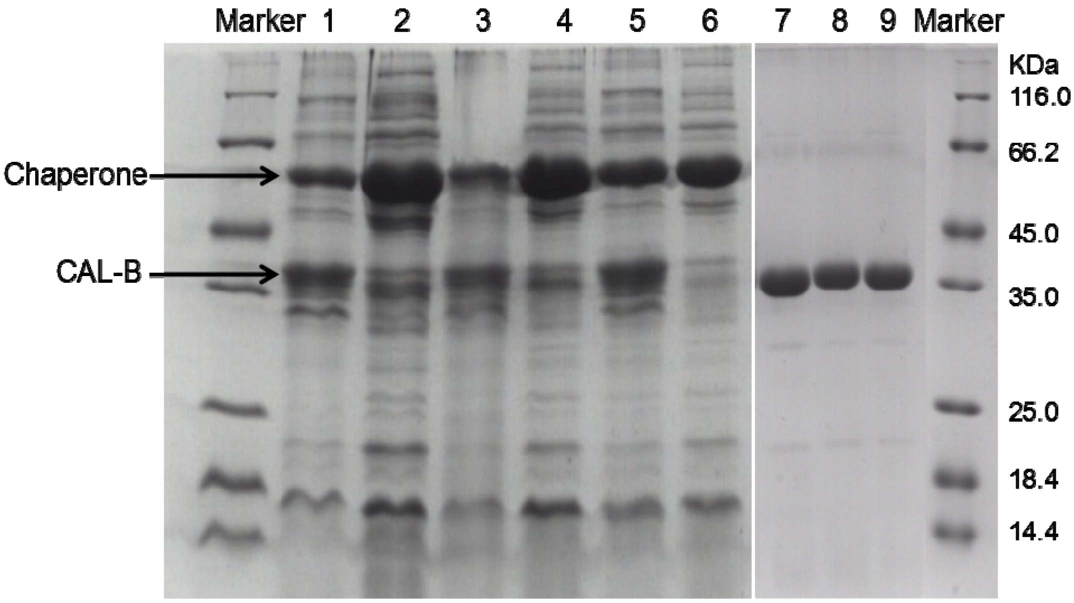

Supplementary Figure 2. SDS-PAGE of WT-CALB and mutants. Lane 1, 3 and 5 are the cellular insoluble fraction of WT-CALB, QW1 and QW4 respectively. Lane 2, 4 and 6 are the cellular soluble fraction of WT-CALB, QW1 and QW4 respectively. Lane 7, 8 and 9 are the purified proteins of WT-CALB, QW1 and QW4 respectively. Source data are provided as a Source Data file.

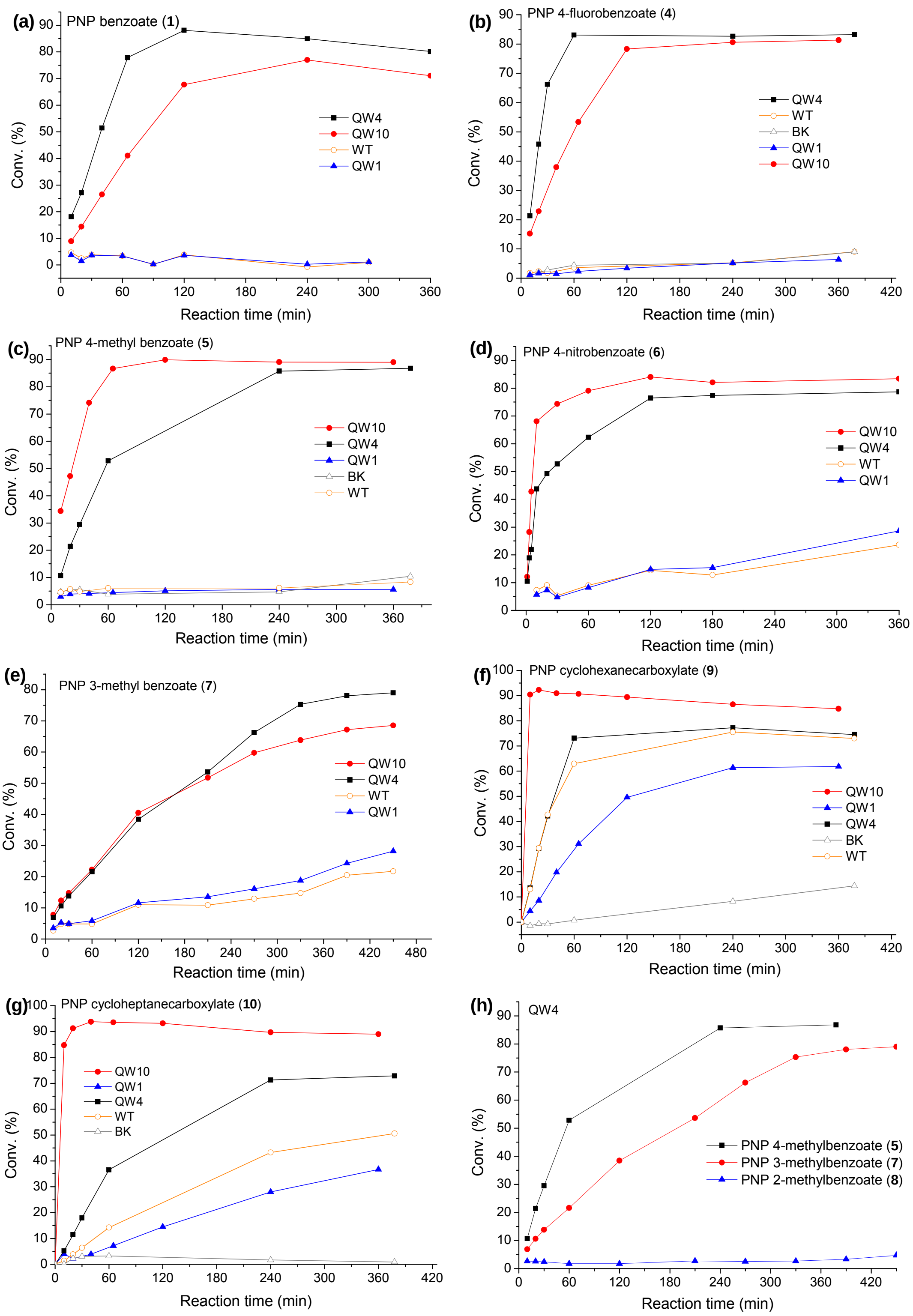

Supplementary Figure 3. The hydrolytic reaction time course of various substrates. **a**, *p*NP benzoate (**1**). **b**, *p*NP 4-fluorobenzoate (**4**). **c**, *p*NP 4-methylbenzoate (**5**). **d**, *p*NP 4-nitrobenzoate (**6**). **e**, *p*NP 3-methylbenzoate (**7**). **f**, *p*NP 2-methylbenzoate (**8**). **g**, *p*NP cycloheptanecarboxylate (**10**). **h**, QW4. Reaction conditions: the solutions of substrates (**1**, **4-10**) in acetonitrile (30 μL, 10 mg/mL), enzymes solution (200 μL, 0.2 g cell/mL), potassium phosphate buffer (370 μL, 50 mM, pH 7.5, 1.0% Triton X100), 37 °C. . Source data are provided as a Source Data file.

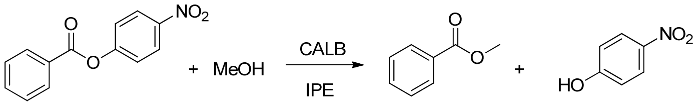

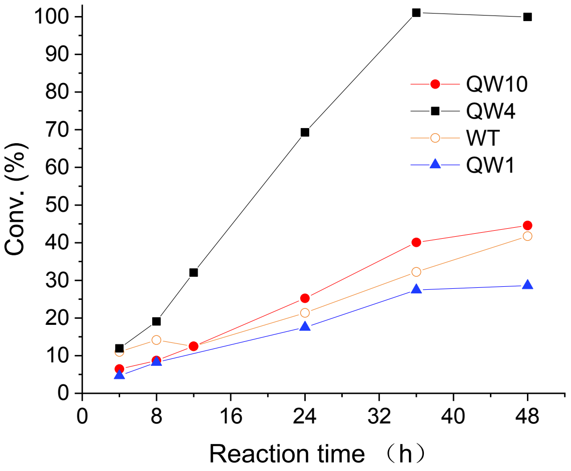

Supplementary Figure 4. The time course of the transesterification of **1** using methanol in organic solvent. Reaction conditions: 10 mg immobilized enzymes (10% enzyme on acrylic resin), 0.5 mL IPE, **1** (40mM) and methanol (5 uL), 50 °C. Source data are provided as a Source Data file.

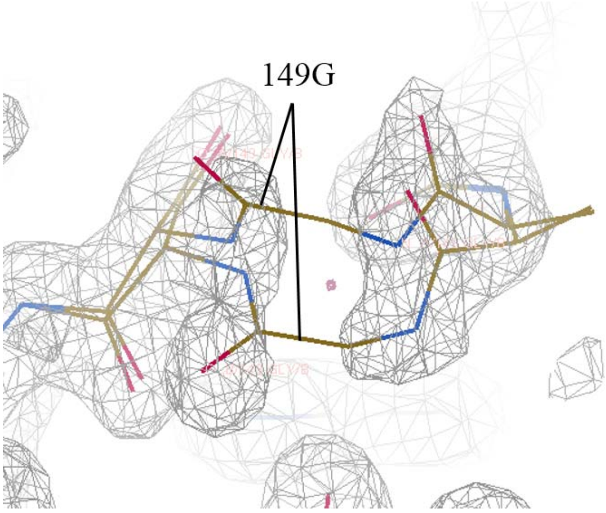

Supplementary Figure 5. Stereoview of the Gly149 region in QW4 mutant. $2F_o$-F_c Electron density map was contoured at 1σ.

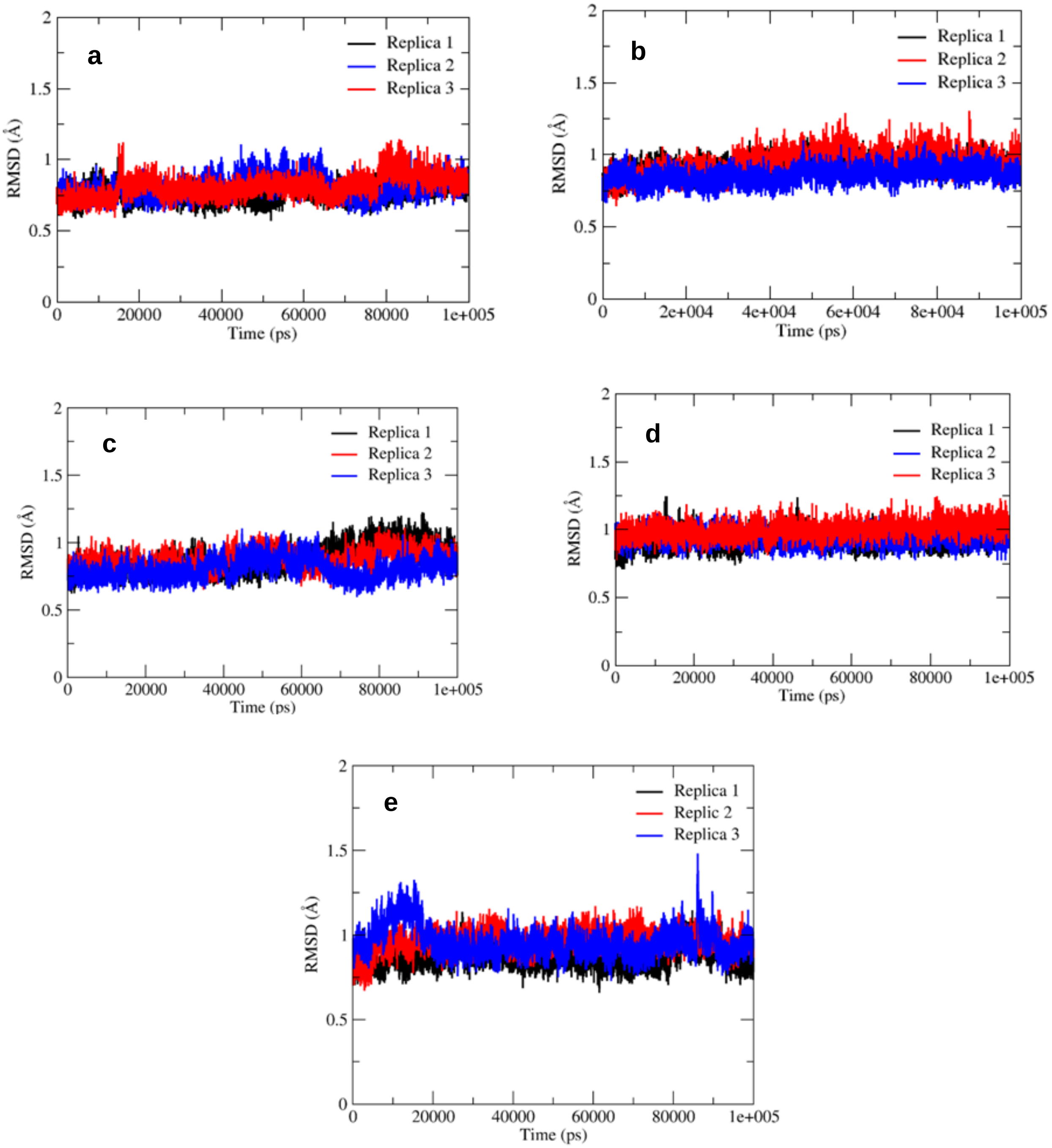

Supplementary Figure 6. RMSD of Cα atom of the protein over 100 ns MD trajectory. **a,** WT CALB. **b,** QW1 (S105C variant). **c,** QW2. **d,** QW10. **e,** QW4.

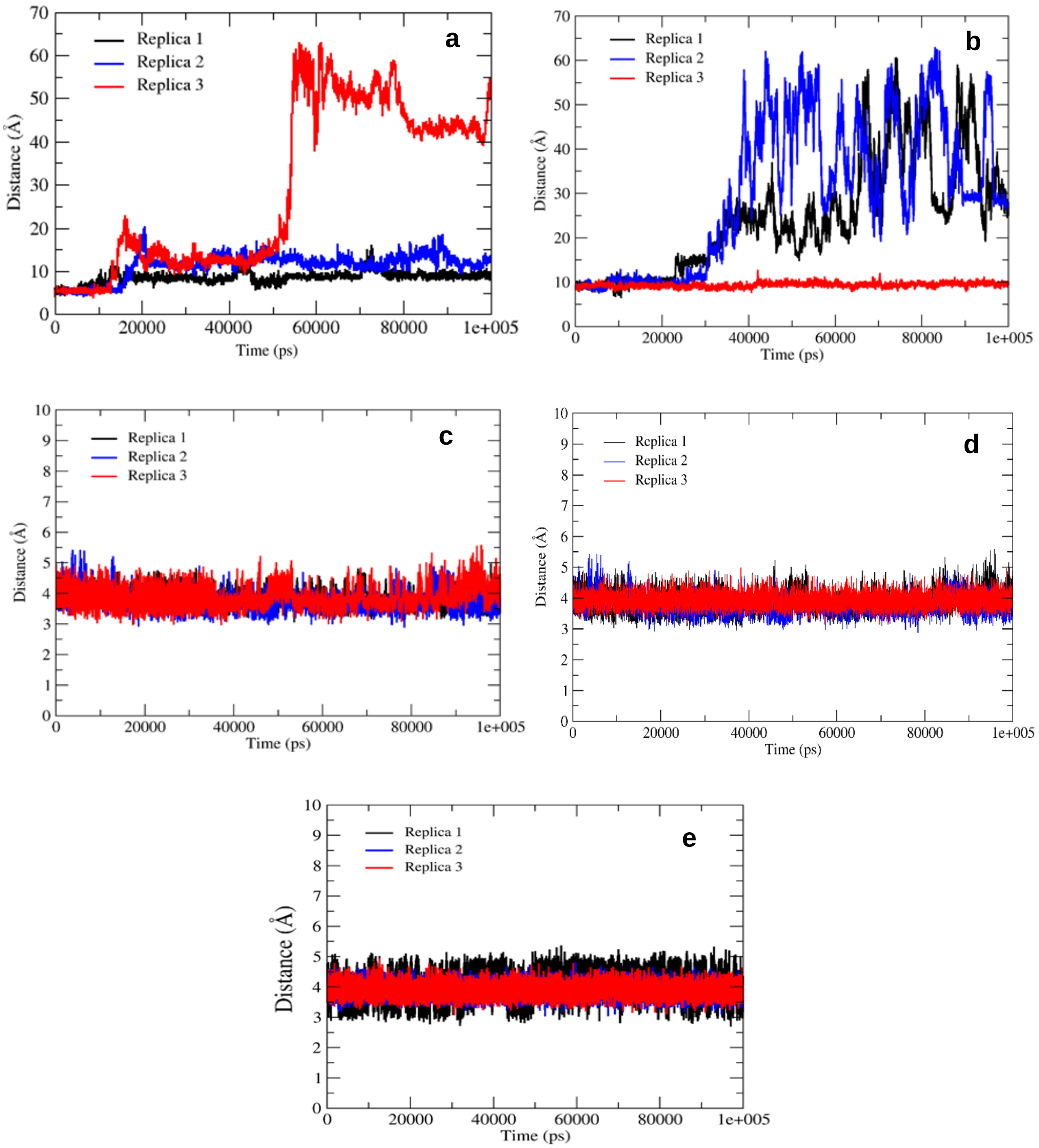

Supplementary Figure 7. The distance of the nucleophilic addition measured over 100 ns MD trajectory. **a,** WT CALB. **b,** QW1 (S105C variant). **c,** QW2. **d,** QW10. **e,** QW4. The distance is between the hydroxyl group of Ser105 (or thiol group of Cys105) and carbonyl carbon atom of the substrate.

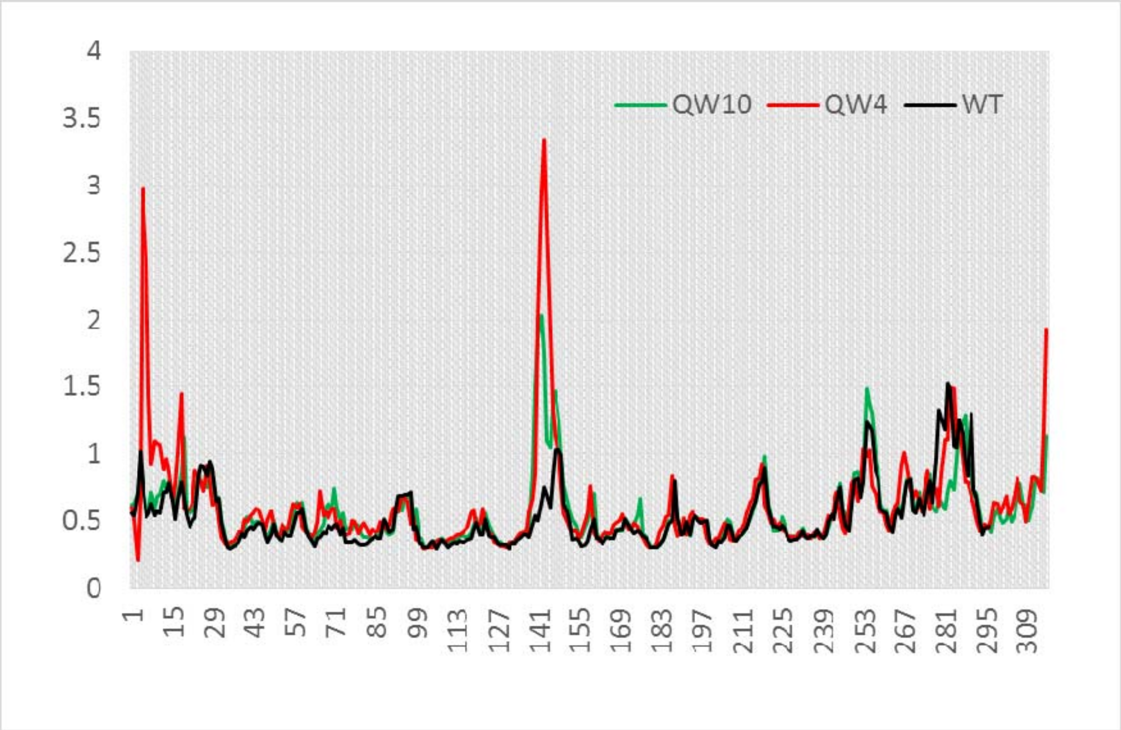

Supplementary Figure 8. RMSF plot of the WT CALB and variants based on 100-ns MD simulations. Loop 137-150 of QW4 and QW10, the lid at the entrance of catalytic sites in CALB, is very flexible as compared to those in WT.

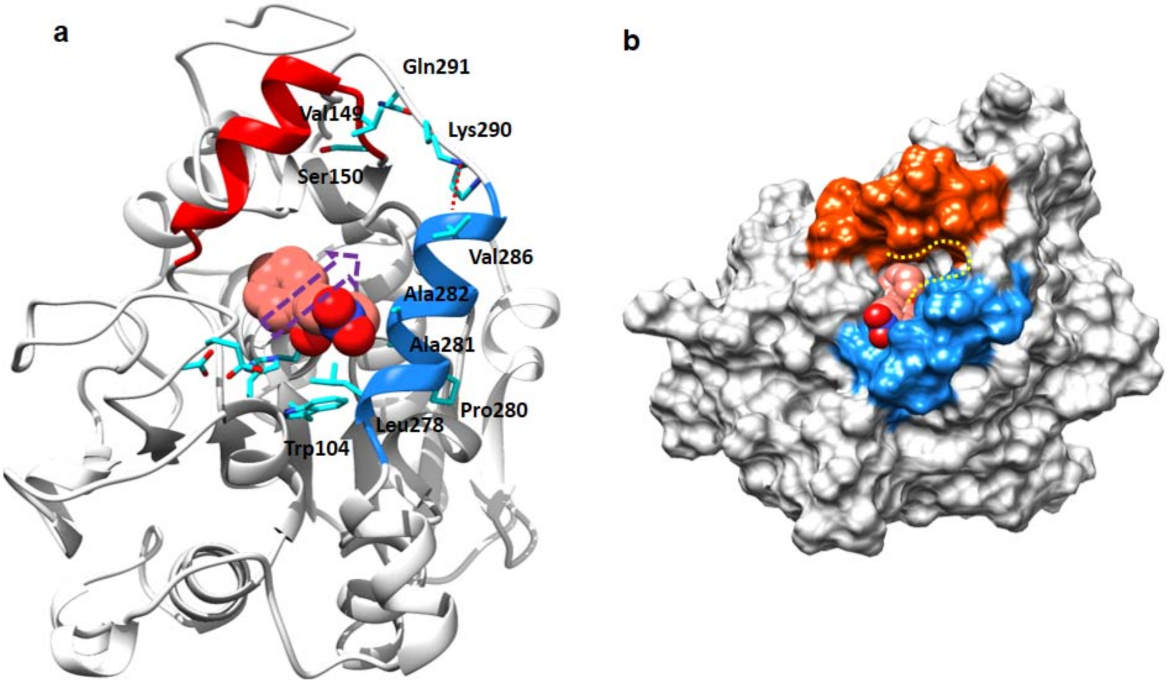

Supplementary Figure 9. Substrate **1** in WT CALB during the initial equilibrated MD trajectory. **a,** The binding modes of the substrate **1** in WT CALB during the initial equilibrated MD trajectory. **b,** Surface representation of the substrate **1** in WT CALB during the initial equilibrated MD trajectory. Loop 137-150 is shown in red cartoon and surface. Helix 277-288 is shown in blue cartoon and surface. Substrate **1** is shown in red sphere.

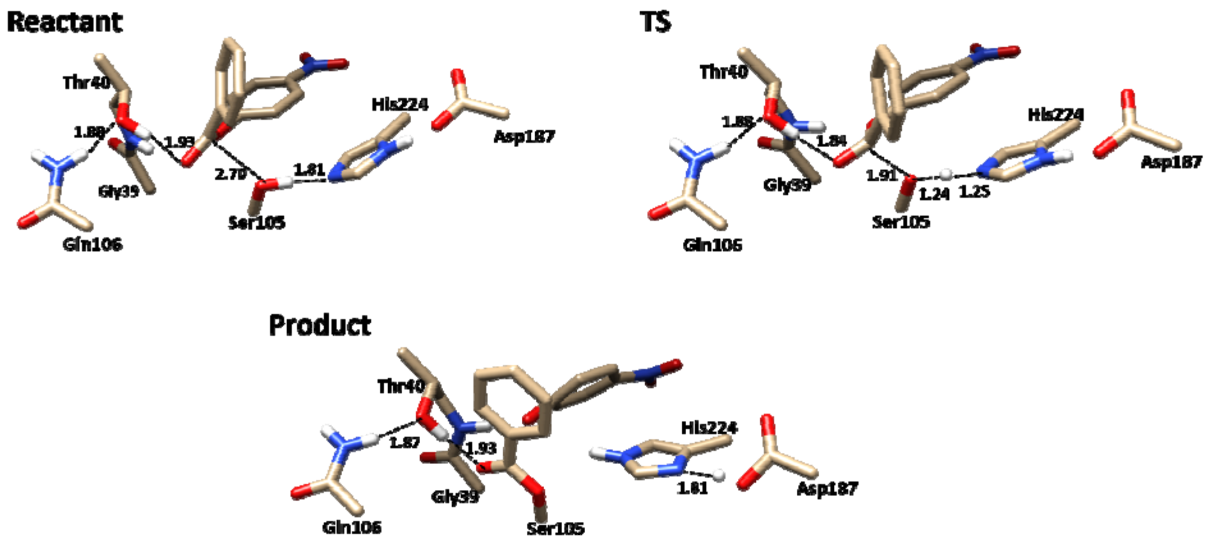

Supplementary Figure 10. The stationary points of the QW10 variant calculated by QM/MM. QM/MM calculations were run using DFT with ωB97X-D functional and 6-31+G (d,p) basis set. The coordinates of QM/MM stationary points of QW10 mutants are in the Supplementary Dataset 2. Formation of the tertiary intermediate occurs via a concerted mechanism where the proton transfer from Ser105 to His224 occurs simultaneously with the nucleophilic addition to the carbonyl carbon atom of the substrate.

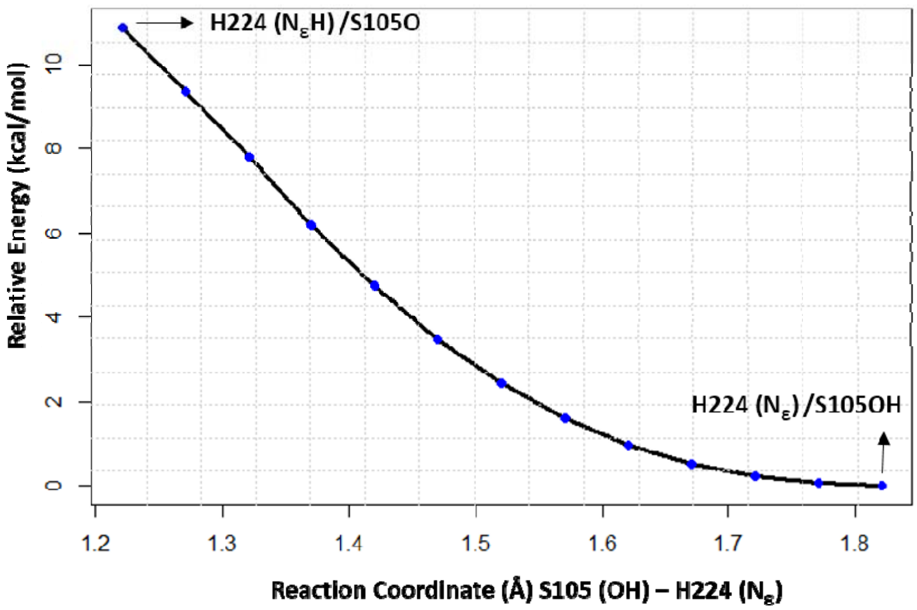

Supplementary Figure 11. Potential surface scan of QW10. The reaction coordinate is S105(OH)-H224 (Nε) corresponding to the proton transfer from the hydroxyl of S105 to the epsilon nitrogen of the H224. QM/MM calculations were run at ωB97X-D/6-31+G (d,p) level. Source data are provided as a Source Data file.

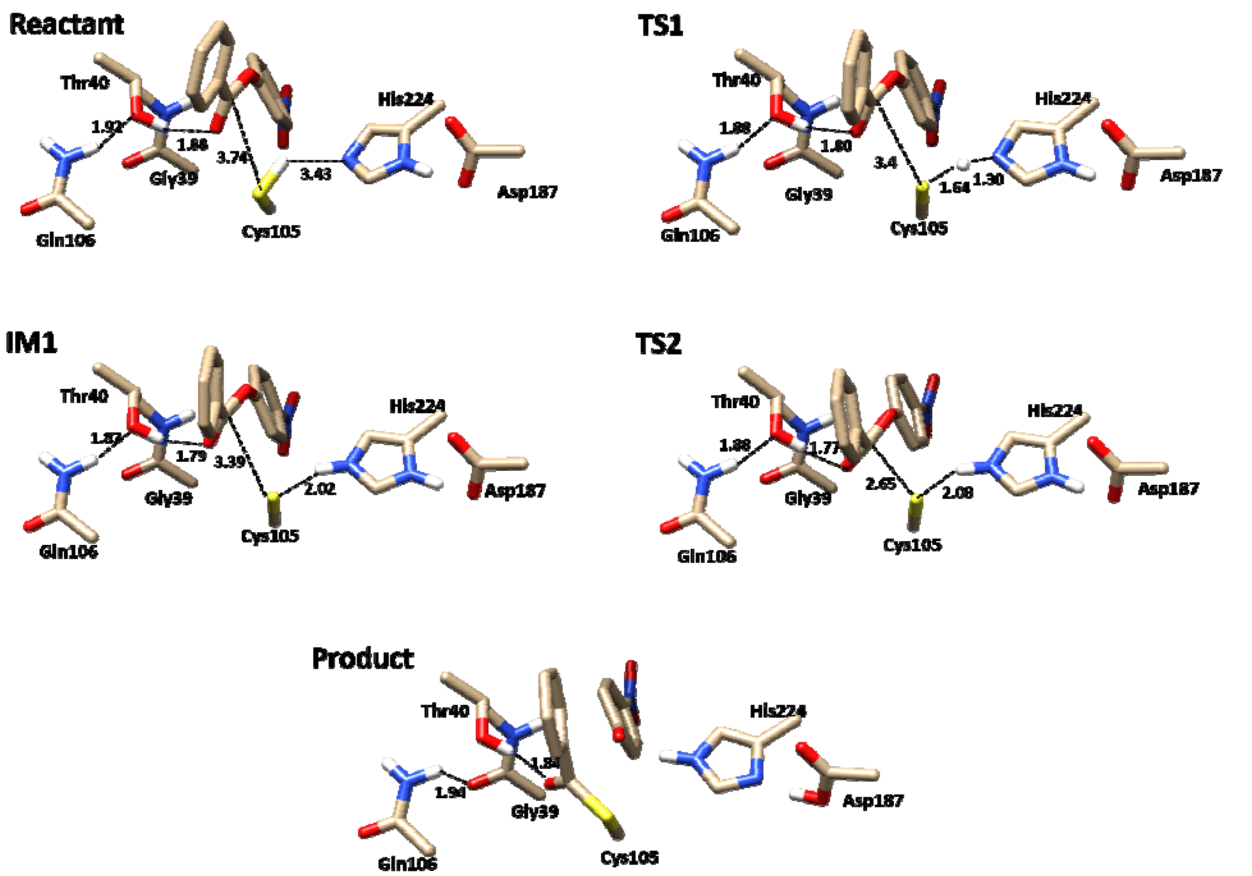

Supplementary Figure 12. The stationary points for QW4 variant calculated by QM/MM. Formation of the tertiary intermediate occurs via a two-step reaction mechanism where proton transfer from Cys105 to His224 is followed by a nucleophilic addition of the deprotonated Cys105 to the carbonyl group of the substrate. QM/MM calculations were run at ωB97X-D/6-31+G (d,p) level. The coordinates of QM/MM stationary points of QW4 mutants are in the Supplementary Dataset 2.

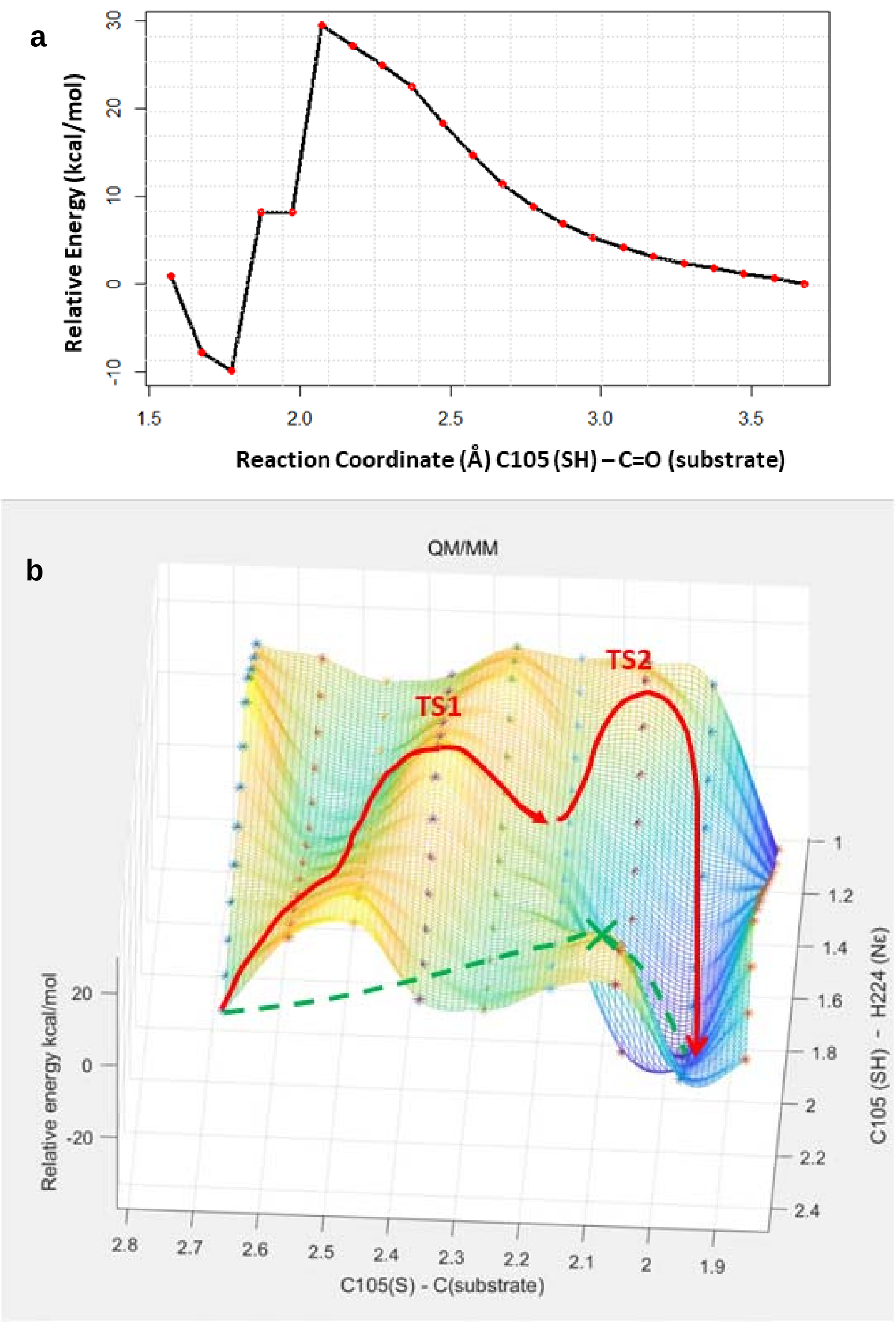

Supplementary Figure 13. Potential energy surface scan for the mutant QW4 in quest for concerted mechanism. **a,** 1D scan: The reaction coordinate is C105(S)-C=O (substrate); **b,** 2D scan: The two reaction coordinates are C105(SH)-H224(Nε) and C105(S)-C=O (substrate). QM/MM calculations are at B3LYP/6-31+G (d,p) level. TS1 and TS2 associated with two-step mechanism are labeled in red, and the potential TS corresponding to the concerted mechanism is marked by green. Optimization of the structure closest to a potential transition state corresponding to the concerted mechanism always led to the $Cys105^{-}/His224^{+}$ zwitterionic pair, indicating that the concerted mechanism is not favorable. Source data are provided as a Source Data file.

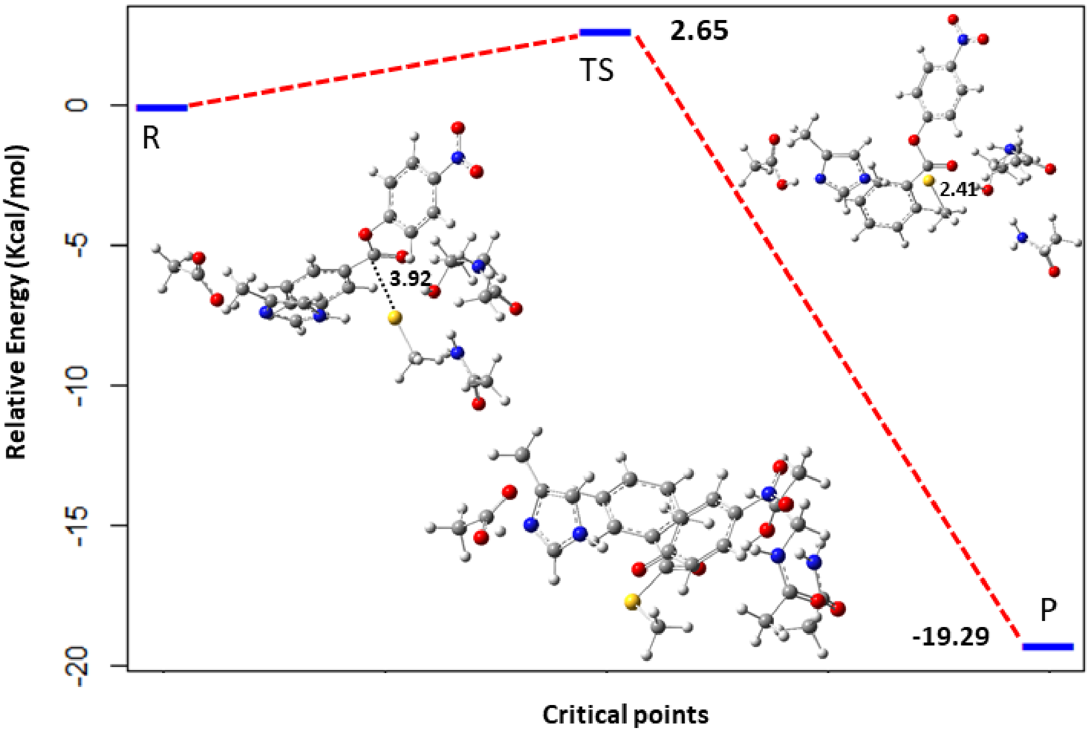

Supplementary Figure 14. The reaction profile for the mutant QW4 calculated by QM cluster model. The reaction coordinate is the distance between the substrate carbonyl carbon and thiolate sulphur of deprotonated Cys105. The QM cluster calculation is at B3LYP/6-31+G (d,p) level.

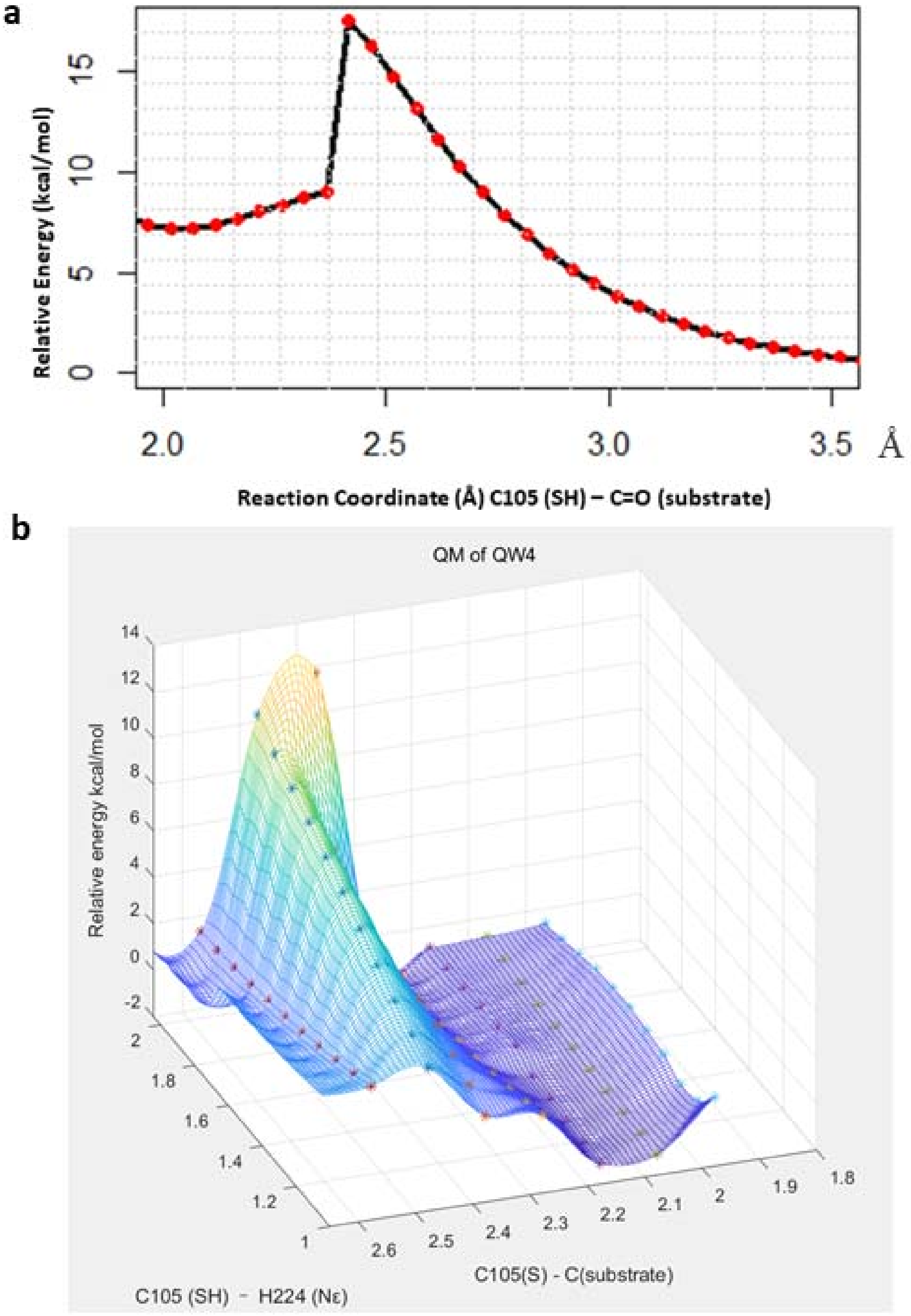

Supplementary Figure 15. Potential surface scan for the mutant QW4 by QM cluster model. **a,** 1D scan: The reaction coordinate is C105(S)-C (Substrate); **b,** 2D scan: The two reaction coordinates are C105(SH)-H224(Nε) and C105(S)-C (Substrate). The QM cluster calculation is at B3LYP/6-31+G (d,p) level. No transition state is located from either scan indicate the concerted mechanism with the proton transfer from Cys105 to His224 concurring with attack of the carbonyl group of the substrate by sulphur atom of Cys105 is not liable. Source data are provided as a Source Data file.

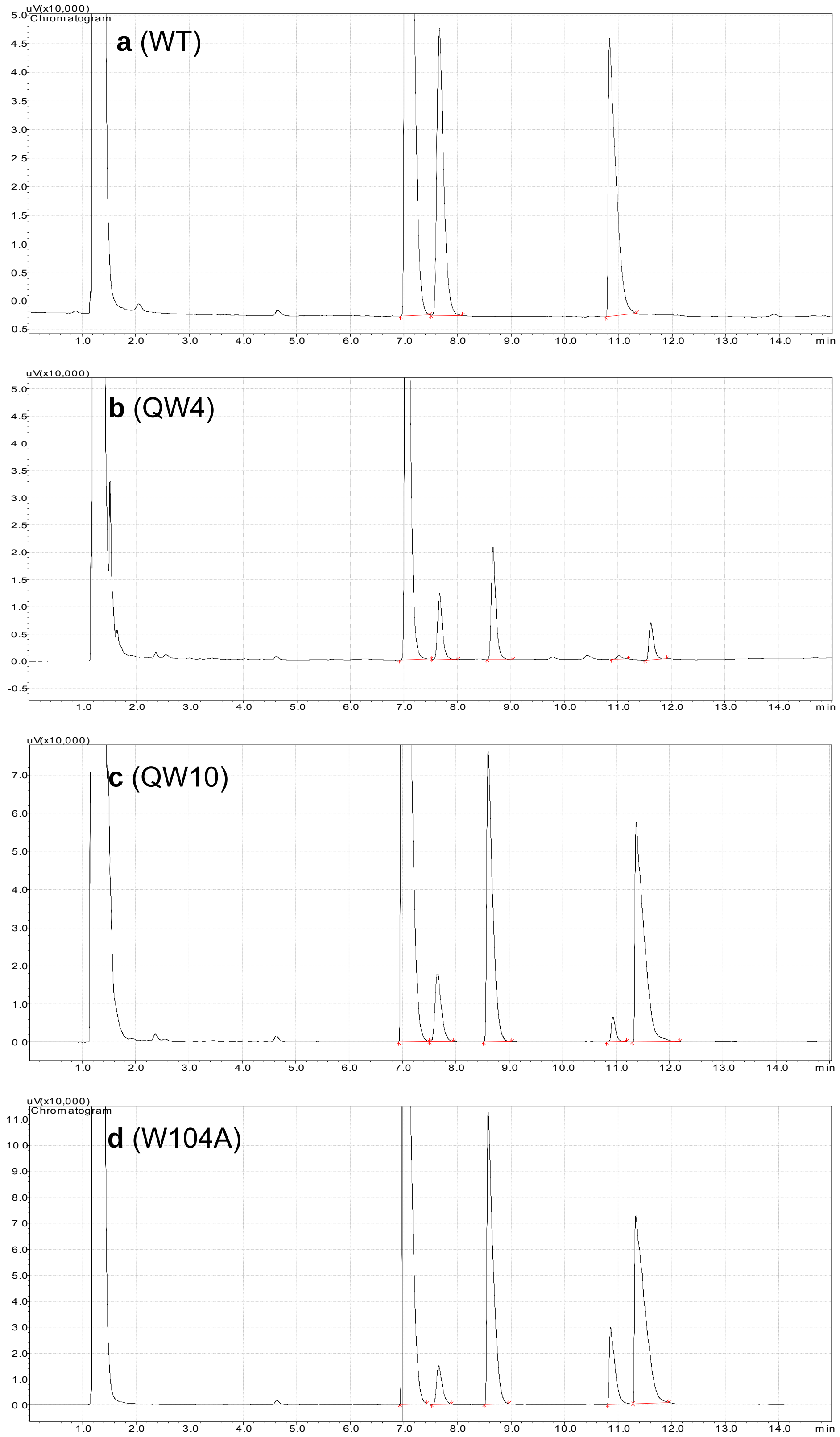

Supplementary Figure 16. Chiral GC of stereoselective hydrolysis of *rac*-**11**. **a**, WT. **b**, QW4. **c,** QW10. **d**, W104A. $T_{(S\text{-}\mathbf{11})}$ = 7.7 min, $T_{(R\text{-}\mathbf{11})}$ = 8.7 min, $T_{(R\text{-}\mathbf{12})}$ = 10.8 min, $T_{(S\text{-}\mathbf{12})}$ = 11.5 min, dodecane (T = 6.9 min) as internal standard. GC conditions: Agilent CP-Chirasil-Dex CB column, initial temperature: 100 ^{o}C, 2 ^{o}C /min heated to 130 ^{o}C, holding 5 min.

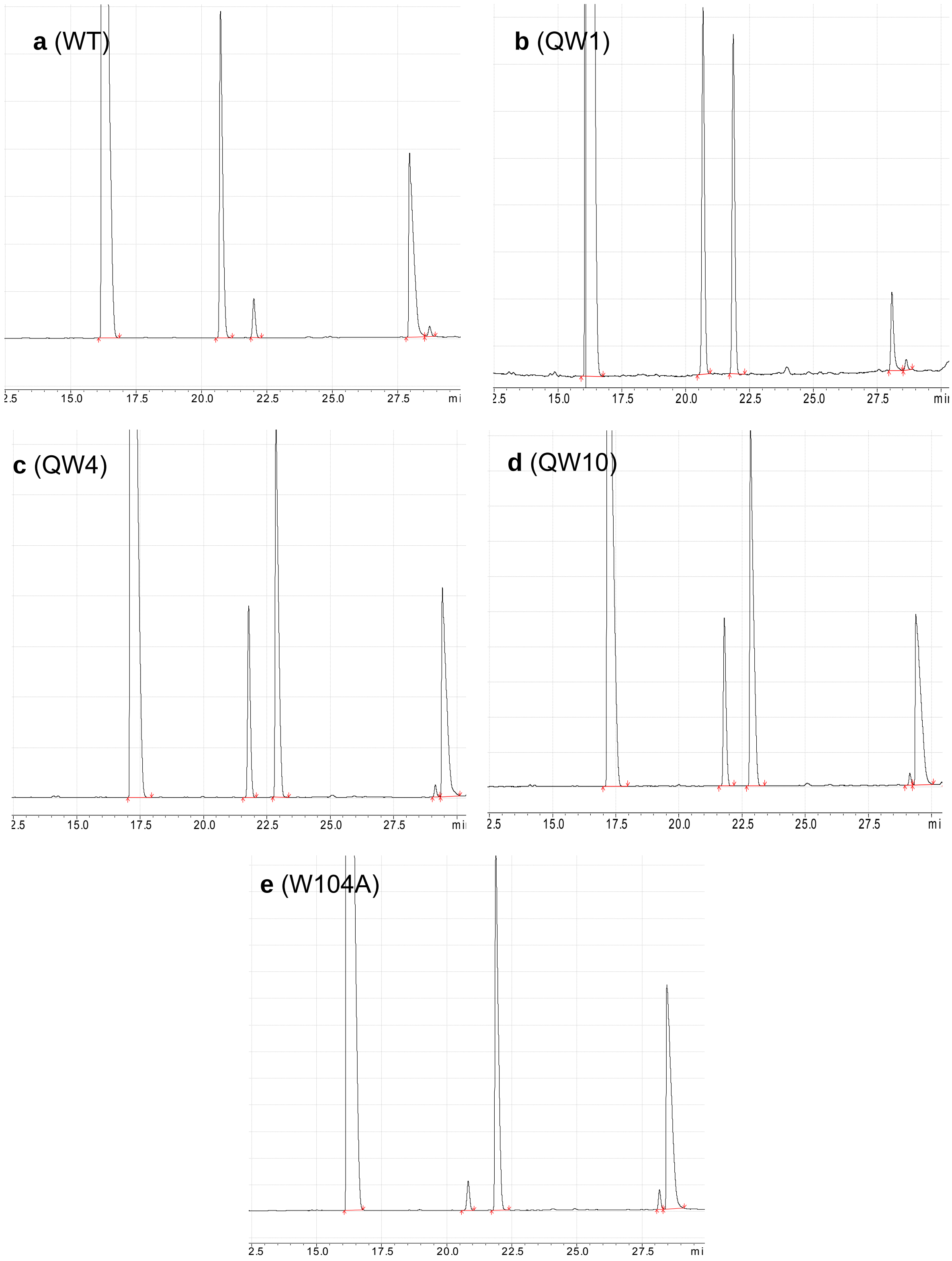

Supplementary Figure 17. Chiral GC of stereoselective hydrolysis of *rac*-**13**. **a**, WT. **b**, QW1. **c,** QW4. **d**, QW10, **e**, W104A. $T_{(S\text{-}\mathbf{13})}$ = 20.9 min, $T_{(R\text{-}\mathbf{13})}$ = 22.0 min, $T_{(R\text{-}\mathbf{14})}$ = 28.1 min, $T_{(S\text{-}\mathbf{14})}$ = 28.6 min, dodecane (T = 16.5 min) as internal standard. GC conditions: Agilent CP-Chirasil-Dex CB column, initial temperature: 70 °C, 2 °C /min heated to 130 °C, holding 5 min.

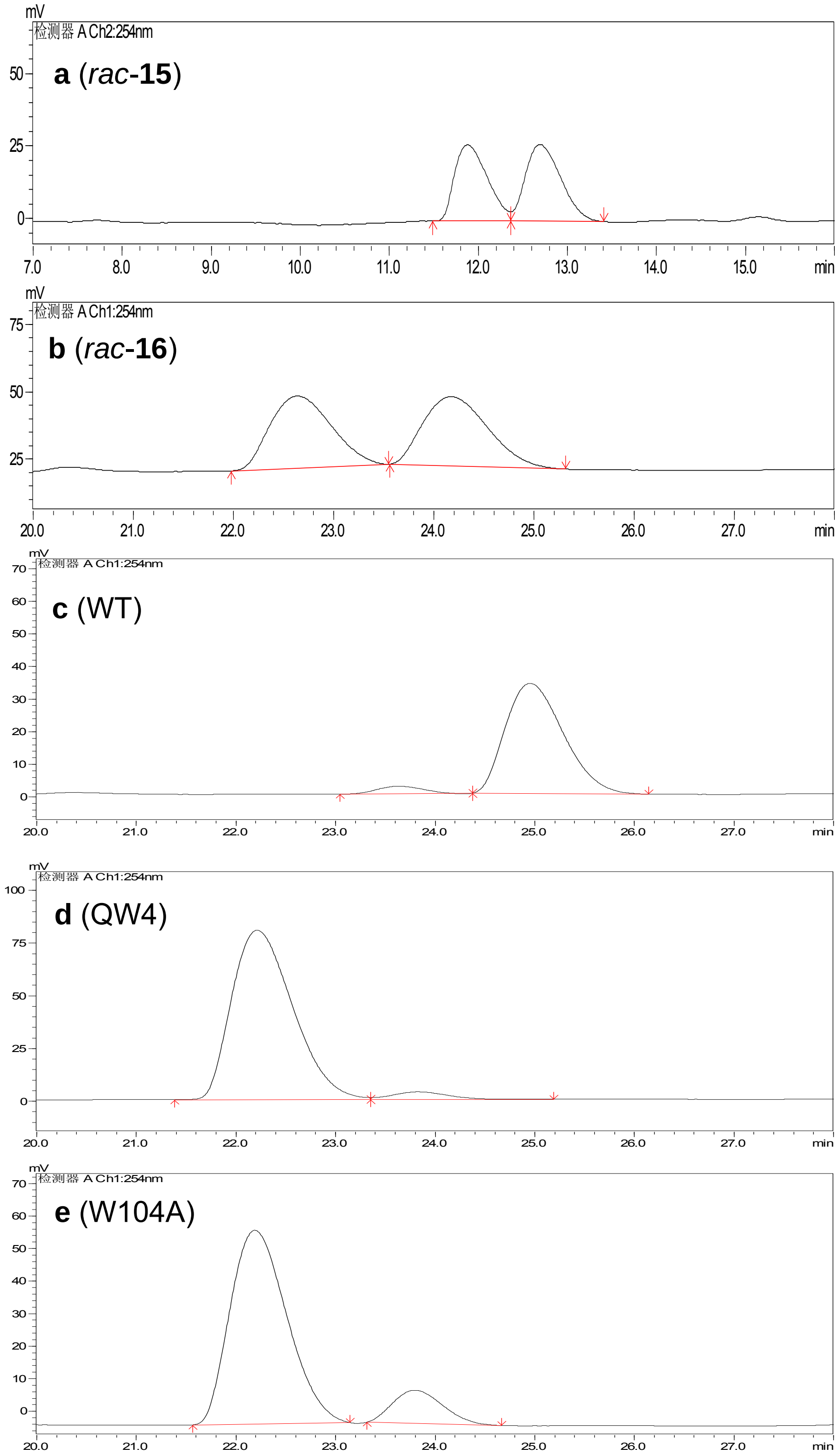

Supplementary Figure 18. Chiral HPLC of stereoselective hydrolysis of *rac*-**15. a,** *rac*-**15**, T_R = 11.8 min, T_S = 12.8 min. **b,** *rac*-**16**, T_S = 22.6 min, T_R = 24.2 min. **c,** WT CALB-catalyzed (*R*)-selective hydrolysis of *rac*-**15**. **d,** QW4-catalyzed (*S*)-selective hydrolysis of *rac*-**15**. **e,** W104A-catalyzed (*S*)-selective hydrolysis of *rac*-**15**. Separation conditions: chiral HPLC, OJ-H, hexane:isopropanol = 98:2 (v:v), λ=254nm, 0.5 ml/min.

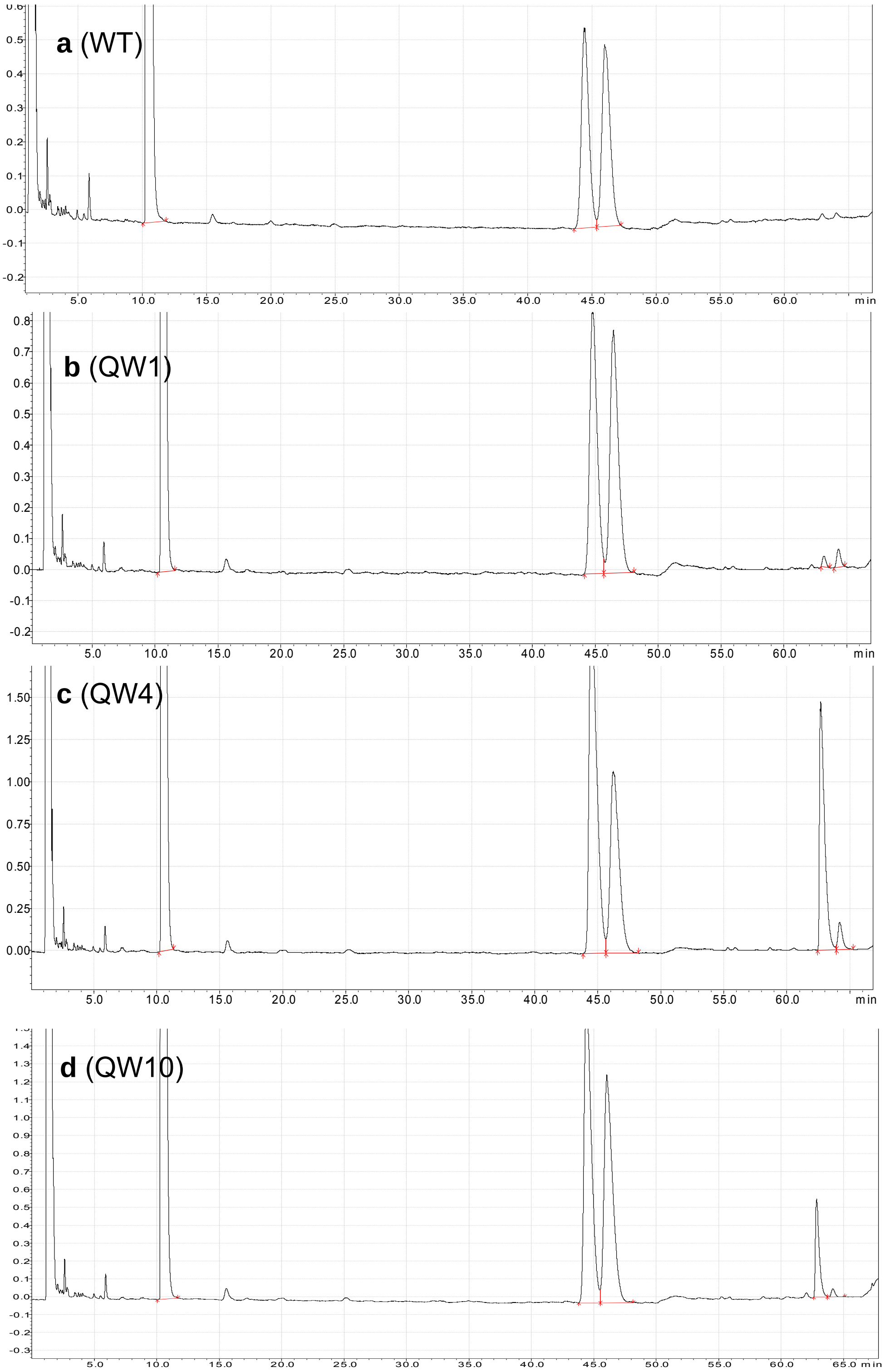

Supplementary Figure 19. Chiral GC of stereoselective hydrolysis of *rac*-**17**. **a**, WT. **b**, QW1. **c,** QW4. **d**, QW10. $T_{(R\text{-}\mathbf{17})}$ = 44.0 min, $T_{(S\text{-}\mathbf{17})}$ = 45.6 min, $T_{(S\text{-}\mathbf{18})}$ = 63.0 min, $T_{(R\text{-}\mathbf{18})}$ = 64.3 min, dodecane (T = 10.3 min) as internal standard. GC conditions: Agilent CP-Chirasil-Dex CB column, initial temperature: 98 ^{o}C, holding 50min, 20 ^{o}C /min heated to 125 ^{o}C, holding 15 min, then 25 ^{o}C /min up to 200 ^{o}C, holding 1 min.

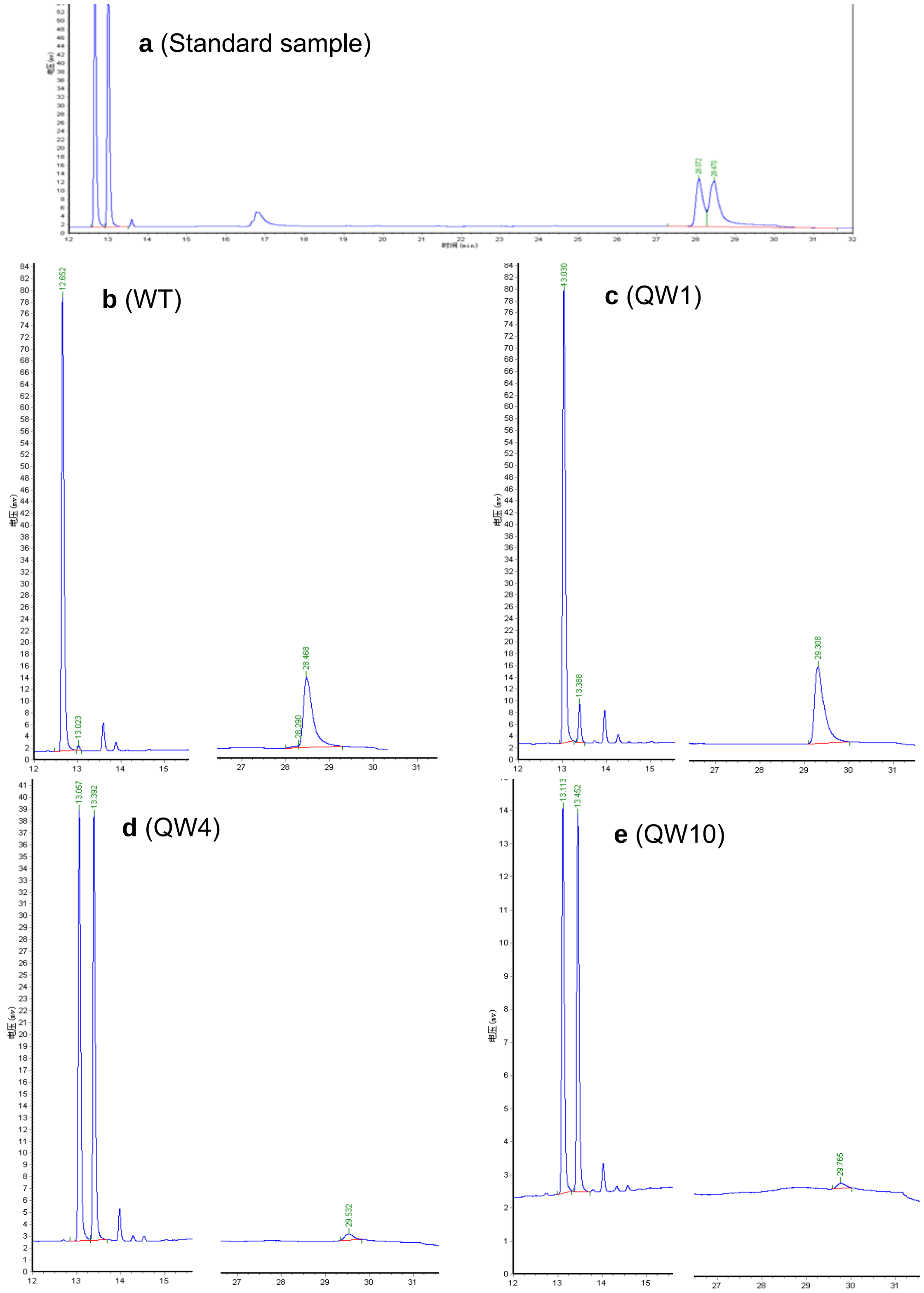

Supplementary Figure 20. Chiral GC of stereoselective hydrolysis of *trans*-**21**. **a,** Standard samples of *trans*-**21** and *trans*-**22**, $T_{(trans\text{-}1S,2S\text{-}\mathbf{21})}$ = 12.65 min, $T_{(trans\text{-}1R,2R\text{-}\mathbf{21})}$ = 12.99 min, $T_{(trans\text{-}1S,2S\text{-}\mathbf{22})}$ = 28.07 min, $T_{(trans\text{-}1R,2R\text{-}\mathbf{22})}$ = 28.47 min; **b,** WT; **c,** QW1; **d,** QW4; **e,** QW10. GC conditions: Agilent CP-Chirasil-Dex CB column, initial temperature: 100 ^{o}C, holding 3 min, 5 ^{o}C /min heated to 160 ^{o}C, holding 16 min. The absolute configuration of substrate and product was confirmed by comparison with chiral standard.

Supplementary Tables

Supplementary Table 1. Summary of nucleophile interconversion of protease and lipase

Enzyme	Type	Active site	Nucleophile exchange	Residual activity relative to WT	Ref
Subtilisin	Serine protease	S221-H64-D32	S221C	10^3 to 10^4-fold less than WT	1, 2
Trypsin	Serine protease	S195-H57-D102	S195C	10^5 to 10^6-fold less than WT	3, 4
Penicillin acylase	Serine protease	S290-H-D	S290C	0.02%	5
pBR322 ß-lactamase	Serine protease	S70-H-D	S70C	3%	6
Myristoyl-ACP thioesterase from *Vibrio harveyi*	Serine protease	S114-H241-D211.	S114C	1%	7
Sindbis virus capsid protein autoprotease	Serine protease	S215-H141-D147	S215C	60%	8
Thioesterase in chicken fatty acid synthase	Serine protease	S101-H-D	S101C	50%	9
Papain	Cysteine protease	C25-H159-N175	C25S	No activity	10
Poliovirus protease 3C	Cysteine protease	C147-H40-E71	C147S	<1%	11
Lantibiotic protease	Cysteine protease	C12-H90-D106	C12S	No activity	12
Hepatitis a virus protease 3C	Cysteine protease	C172-H44-D98	C172S	No activity	13
Tobacco etch virus (TEV) protease	Cysteine protease	C151-H-D	C151S	<0.01%	14
			$TEV^{Ser}X$ mutant containing C151S (obtained after ten rounds of directed evolution)	<10%	
Lecithin retinol acyltransferase	Acyltransferase	C161-H	C161S	3%	15
Bacteriophage lysine PlyC	Peptidoglycan hydrolases	C333-H420	C333S	<5%	16
Staphylococcus hyicus lipase	Lipase	S369-H-D	S369C	0.2%	17
Human lipoprotein lipase	Lipase	S132-H-D	S132C	No activity	18
Ca^{2+}-sensitive cytosolic phospholipase A	Lipase	S228-H-D	S228C	No activity	19
Escherichia coli outer membrane phospholipase A	Lipase	S144-H-D	S144C	1%	20

Supplementary Table 2. Specific activity and sequence of different CAL B mutants. [a]

Library	Enzyme	Specific activity($\mu M \times Min^{-1} \times OD^{-1}$) [b]	Sequence
--	WT	2,7	/
A	QW2	5,9	S105C/W104V
B	QW3	8,7	S105C/W104V/A281Y/A282Y
	B4	8,1	S105C/W104V/A281L/A282W
	B7	6,6	S105C/W104V/A281F/A282F
C	QW4	11,4	S105C/W104V/A281Y/A282Y/V149G
	B9	10,9	S105C/W104V/A281Y/A282Y/V149G/L144C
	B10	10,8	S105C/W104V/A281Y/A282Y/V149G/L144F
	B11	8,8	S105C/W104V/A281Y/A282Y/V149D/L144F
	B12	9,3	S105C/W104V/A281Y/A282Y/V149C/L144Y
	B13	8,6	S105C/W104V/A281Y/A282Y/V149C/L144C
	B14	8,7	S105C/W104V/A281Y/A282Y/V149H/L144F
	B15	10,1	S105C/W104V/A281Y/A282Y/V149D/L144H
	B16	7,8	S105C/W104V/A281Y/A282Y/V149V/L144F
	B17	10,5	S105C/W104V/A281Y/A282Y/V149F/L144F

[a] Source data are provided as a Source Data file. [b] Average of three measurements.

Supplementary Table 3. Stereoselectivity of hydrolytic kinetic resolution of *rac*-**11** - **21**

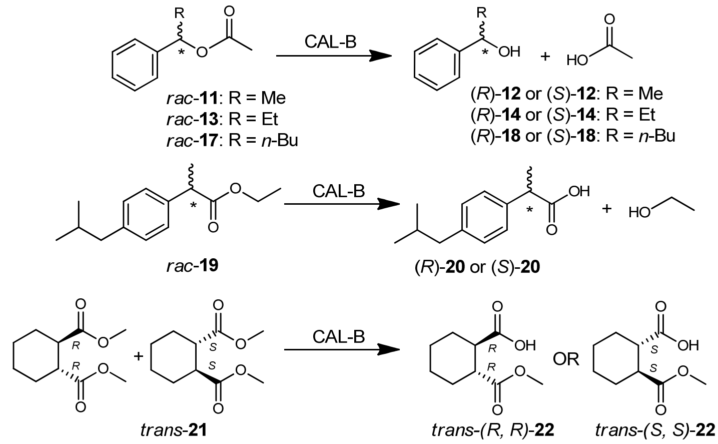

Substrates	Entry	Enzyme	Reaction time (h)	Conv. (%)	ee_p (%)	E
rac-**11**	1	WT	12	49	99	>200 (R)
	2	QW1	48	5	63	5 (R)
	3	QW4	48	22	82	13 (S)
	4	QW10	12	46	90	43 (S)
	5	W104A	9	55	63	10 (S)
rac-**13**	6	WT	5.5	47	93	71 (R)
	7	QW1	48	5	79	9 (R)
	8	QW4	48	31	95	59 (S)
	9	QW10	28	33	96	68 (S)
	10	W104A	4	49	92	70 (S)
rac-**17**	11	WT	72	-	-	1 (R)
	12	QW1	120	5	31	2 (R)
	13	QW4	72	24	82	13 (S)
	14	QW10	72	8	85	13 (S)
rac-**19**	15	WT	2	45	19	2 (R)
	16	QW1	8	13	6	1 (R)
	17	QW4	8	8	14	1 (R)
	18	QW10	8	11	47	3 (R)
**21**	19	WT	6	46	94	80 (trans-1R,2R)
	20	QW1	6	44	99	>200 (trans-1R,2R)
	21	QW4	6	<5	99	--
	22	QW10	6	<5	99	--

Supplementary Table 4. X-ray data collection and refinement statistics

**Name**	QW4(oxidized)	QW4(unoxidized)	QW10
**PDB ID**	6ISQ	6ISR	6ISP
**Data collection**			
Space group	$P2_1$	$P2_1$	$P2_1$
Cell dimensions			
a, b, c (Å)	44.7, 132.4, 52.4	52.3, 44.6, 132.9	47.0, 92.5, 156.0
α, β, γ (°)	90.0, 90.037, 90.0	90.0, 89.4, 90.0	90.0 90.013, 90.0
Resolution(Å)	50 -1.86 (1.89 - 1.86)	50 - 2.60 (2.64 - 2.60)	46.26 -1.88 (1.91 - 1.88)
R_{merge}(%)	14.1 (67.5)	20.0 (82.4)	13.2 (99.3)
Completeness(%)	99.7 (98.8)	99.6 (98.2)	98.8 (99.2)
Average(I/σ)	14 (2.0)	8.8 (1.9)	9.4 (2.1)
Redundancy	5.5	4.5	6.6
Wilson B-factor(Å^2)	18.74	42.43	23.67
**Refinement**			
Resolution(Å)	37.04 - 1.85	42.25 - 2.58	42.26 - 1.88
No. reflections uesd	50329	17958	106351
R_{work} / R_{free}	0.1601 / 0.1893	0.1800 / 0.2315	0.2135 / 0.2383
Asymmetric unit	2	2	4
No. non-H atoms			
Protein	4796	4625	9336
Ligand	48	49	344
Water	300	57	1228
Overall B factor(Å^2)	21.2	41.6	25.6
Ramachandran			
Favored (%)	97.71	96.0	96.36
Outliers (%)	0.31	0.00	0.16
rotamer outliers (%)	0.38	0.20	0.29
RMSD			
Bond lengths(Å)	0.018	0.007	0.003
Bond angles (°)	1.483	0.869	0.924

Supplementary Table 5. List of forward primers

Library	Primer	Sequence [a]
A	For W104/S105 NDT	*GCTTCCCGTGCTCACC**NDTNDT**CAGGGTGGTCTGG*
B	For A281/A282 NDT	*CCTGGCGCCG**NDTNDT**GCAGCCATCGTGGCGGGTCCAAAGC*
C	For L144/V149NDT	*CGCCGGCCCT**NDT**GATGCACTCGCG**NDT**AGTGCACCC*
D	For I189/V190 NDT	*GGCGACCGACGAG**NDTNDT**CAGCCTCAGGTGTCC*
E	For L140/A141 NDT	*GGCACCGTC**NDTNDT**GGCCCTCTCGATGCACTCG*
F	For L278/I285NDT (281Y282Y)	*CGGCTGCGCTC**NDT**GCGCCGTATTATGCAGCC**NDT**GTGGCGGG*
	For S105C	*CCCGTGCTCACCTGG**TGT**CAGGGTGGTCTGG*
	Silent reverse primer	*GGGAGCAGACAAGCCCGTCAGGG*

[a] Degenerate nucleotide designation; N=A, C, G, T; D=A, G, T; T=T. Primers were ordered from Eurofins MWG Operon.

Supplementary Methods

Chemicals.

p-Nitrophenyl benzoate (**1**), *p*-nitrophenyl 4-fluorobenzoate (**4**), *p*-nitrophenyl 4-methylbenzoate (**5**), *p*-nitrophenyl 4-nitrobenzoate (**6**), *p*-nitrophenyl 3-methylbenzoate (**7**), *p*-nitrophenyl 2-methylbenzoate (**8**), *p*-nitrophenyl cyclohexanecarboxylate (**9**), and *p*-nitrophenyl cycloheptanecarboxylate (**10**) were synthesized according to the literature method[21-22]. 1-Phenylethyl acetate (*rac*-**11**), 1-phenylpropyl acetate (*rac*-**13**), 1-phenylbut-3-en-1-yl acetate (*rac*-**15**), and 1-phenylpentyl acetate (*rac*-**17**) were synthesized by literature methods[23-24]. All other compounds were obtained from commercial suppliers and used without further purification.

Library Generation.

Saturation mutagenesis libraries were constructed by the QuikChange™ protocol using an improved PCR-based method at sites A (Trp104/Ser105), B (Ala281/Ala282), C (Leu144/ Val149), D (Ile189/Val190), E (Leu140/Ala141), and F (Leu278/Ile285).[25-26] PCR reactions were performed using WT-CALB plasmid (pETM11-CALB) as the template DNA, and forward primers (see Supplementary Table 5) and a silent reverse primer (*GGGAGCAGACAAGCCCGTCAGGG*, 2444–2466 bp of pETM11). The reaction (100 µL final volume) contained: 10 × KOD buffer (10 µL), $MgCl_2$ (4 µL, 25 mM), dNTP (10 µL, 2 mM each), forward primers (4 µL, 2.5 µM each), silent reverse primer (4 µL, 2.5 µM each), template plasmid (1 µL, 100 ng μL^{-1}) and 1 µL of KOD polymerase. PCR conditions used were 95 °C, 3 min; five cycles of (98 °C, 1 min; 65 °C, 1 min; 72 °C, 5 min) for the generation of megaprimer; 20 cycles of (98 °C, 1 min; 72 °C, 8 min); and final extension at 72 °C, 10 min. The initial template was digested by Dpn I (New England Biolabs): 20 µL of PCR reaction mixture were mixed with 1 µL Dpn I (10 U/µL) and incubated overnight at 37°C, followed by an additional 1 µL of Dpn I for 3 h. The digested mixture was purified by QIAquick PCR purification kit, and was then used to transform into electro-competent cells of *E. coli* BL21(DE3) (containing chaperone plasmid pGro7, Takara, Japan). The transformation mixture was incubated with 800 µL of LB medium at 37°C with shaking and spread on LB-agar plates containing 34 µg/mL Kanamycin and 34 µg/mL Chloramphenicol.

Expression and Screening of Library.

Transformants grown on LB-agar plates were picked and cultured overnight at 37 °C with shaking (800 rpm) in 96 deep well plates (capacity, 2.2 mL; Thermo Scientific, UK) containing 800 µL TB with 34 µg/mL Kanamycin and 34 µg/mL Chloramphenicol. An aliquot of 100 µL of culture was used for glycerol stock plates and stored at −80 °C. Then 100 µL of the same culture was transferred into 96 deep well plates containing fresh 700 µL TB, 34 µg/mL Kanamycin, 34 µg/mL Chloramphenicol, and L-arabinose (1mg/mL, the inducer for the expression of chaperone pGro7). The plates were shaken at 37 °C for 4 h, and then cooled to room temperature for 1h. Then isopropyl β-thiogalactopyranoside (IPTG) with the final concentration of 1 mM was used to induce the expression of CALB. After the expression of CALB for 24 h at 18°C, cells were harvested by centrifugation at 2750 ×*g* and 4 °C for 25 min. The cell pellets of each well were resuspended in 400 µL of 50 mM Tris-HCl (pH 8.0) containing 1 mg/mL lysozyme and 4 units of Dnase I. The plates were incubated at 37 °C and 800 rpm for 1 h. Cell debris was precipitated by centrifugation at 2750 ×*g* and 4 °C for 25 min. 120 µL of each cleared supernatant was transferred to a 200 µL 96-well microtiter plate. 10 µL solution of substrate **1** (1mM) diluted from stock solution (20mM in acetonitrile) with phosphate buffer (50 mM potassium phosphate, 1.0% Triton X-100, pH 7.5) was dispensed into the microtiter plate containing

CALB libraries supernatant. The hydrolytic reaction was performed at 37 °C under shaking of 200 rpm. The conversion was evaluated by end-point measurements using a Spectramax Plus384 from Molecular Devices (Sunnyvale, USA) at 405 nm after 30 min reaction time. Positive hits were selected for purification and kinetic measurements, sequenced, and used as DNA template for the next round of saturation mutagenesis.

Enzyme Purification.

200 mL of TB media containing L-arabinose inducer (1mg/mL), Kanamycin (34 μg/mL) and Chloramphenicol (34 μg/mL) in 1-L flask was inoculated with 2 mL overnight culture of the appropriate overexpression strain grown in LB media. The culture was shaken at 37 °C until the optical density at 600 nm reached 0.6, and then cooled in 4°C fridge for 1h. IPTG with a final concentration of 1 mM was added to induce the expression of CALB. After the expression of CALB for 24 h at 18°C, cells were harvested by centrifugation at 4500 ×*g* for 25 min at 4 °C. The cell pellets were resuspended in 5 mL 50 mM Tris-HCl buffer (pH 7.5) and lysed by sonification. Cell debris was precipitated by centrifugation at 20130 ×*g* and 4 °C for 25 min. The supernatant was filtered and loaded on a GE Healthcare HisTrap FF Crude column (5 mL) pre-equilibrated with 50 mM Tris-HCl buffer containing 0.5 M NaCl and 5 mM imidazole. Impurity and pGro7 chaperone (containing GroEL protein with molecular weight about 60 KD) were removed by imidazole at the concentration of about 40 mM and 85 mM respectively, and the purified CALB protein was eluted with the same Tris-HCl buffer containing 100 mM imidazole. The enzyme fraction was desalted and concentrated by using an ultrafiltration centrifugal filter (10 kD cut-off membrane, Amicon), then another ultrafiltration centrifugal filter with 50 kD cut-off membrane was used to cut off the small amount of chaperone pGro7 that remains in the enzyme fraction. The purified enzymes were dissolved by 50 mM Tris-HCl buffer (pH 7.5) and stored at -80 °C. The purity of the enzyme was tested by SDS–PAGE, and the concentration of the purified enzyme was estimated by the Bradford method (Bio-Rad protein assay kit). The protein expression levels of WT CALB, variant QW1, QW2, QW3 and QW4 were approximately 1.23, 0.54, 0.57, 0.89 and 0.43 mg/L, respectively.

Kinetic Measurements of Purified Enzymes.

The kinetics measurements of purified enzymes for substrate **1**, **4-7** were performed at 25 °C in potassium phosphate buffer (50 mM, pH 7.5, 1.0% Triton X-100) at various concentration ranges of substrates. The hydrolytic activities were determined on a Molecular Devices Spectramax (Molecular Devices GmbH, Germany) by monitoring the time-dependent appearance of *p*-nitrophenolate (**3**). The obtained data were fitted to the Michaelis-Menten equation by nonlinear regression analysis.

Hydrolysis Reactions of Substrate 1, 4-10, *rac*-11-21.

In a general protocol of hydrolysis reaction, the solutions of substrates (**1, 4-10,** *rac*-**11,** *rac*-**13,** *rac*-**15,** *rac*-**17,** *rac*-**19,** *trans*-**21**) in acetonitrile (30 μL, 10 mg/mL) and enzymes solution (200 μL, 0.2 g cell/mL) were added to potassium phosphate buffer (370 μL, 50 mM, pH 7.5, 1.0% Triton X100). The reaction mixtures were shaken at 37 °C for the appropriate time. Then the reaction solutions were acidified by adding 10% HCl (20 μL) and extracted with 600 μL dichloromethane and diethyl ether, respectively. Organic phase was evaporated by using Speed-Vac alpha RVC (Christ). 400 μL MTBE containing $C_{12}H_{26}$ as internal standard were added to the tubes. The conversions of substrates **1**, **4-10** were determined by using achiral GC. The substrates of *rac*-**11**, **13**, **17** and **21** were analyzed by chiral GC. The substrates of *rac*-**15** and **19** were analyzed by chiral HPLC. Chromatographic analysis of *rac*-**11**, **13**, **15**, **17** and **21** is

described in the Supplementary Figure 16-20. Absolute stereochemistry was determined by comparison with chiral standards.

Transesterification of *p*-Nitrophenyl Benzoate (1) using Methanol.

10 mg immobilized WT CALB and variants (10% enzyme on acrylic resin) was added to 0.5 mL anhydrous IPE containing substrates **1** (40mM, 4.86mg) and methanol (5 uL), and this mixture was shaken at 200 rpm and 50 °C for different reaction time. The reaction mixture was sampled at different intervals for HPLC analysis. Separation conditions: HPLC, OJ-H, hexane:isopropanol = 10:90 (v:v), λ=254nm, 1 ml/min..

Crystallization and X-ray Structural Analysis.

QW4 was crystalized at same condition inside and outside gloves box, in 0.1 M sodium acetate (pH 6.0), 25% w/v PEG 4000, 8% v/v isopropanol, using the sitting-drop vapor diffusion method at 16 °C. Proteins (at 12 mg/mL) were mixed in a 1:1 ratio with the reservoir solution in a final volume of 4μL. *n*-Dodecyl-N,N-dimethylamine-N-oxide was added to a final concentration of 0.5% w/v. The mix was equilibrated against the reservoir solution. QW10 was crystalized in 0.2 M calcium acetate, 0.1 M HEPES (pH 7.5), 12% w/v PEG 8000, using the sitting-drop vapor diffusion method at 16 °C. Proteins (at 12 mg/mL) were mixed in a 1:1 ratio with the reservoir solution in a final volume of 4μL. N,N-bis-(3-D-Gluconamidopropyl)deoxycholamide was added to a final concentration of 1.4 mM. The mixture was equilibrated against the reservoir solution. All crystals were mounted in nylon loops and flash-frozen in liquid nitrogen. Diffraction data of QW4 (oxidized) was collected at the wavelength of 0.97853 Å in beamline BL19U1 of the National Center for Protein Science Shanghai (China). Diffraction data of QW4 (unoxidized) was collected at the wavelength of 0.97930 Å in beamline BL18U1 of the National Center for Protein Science Shanghai (China). Both data sets were indexed, integrated, and scaled using the HKL3000 package[27]. Diffraction data of QW10 was collected at the wavelength of 0.97918 Å in beamline BL17U1 of Shanghai Synchrotron Radiation Facility. The data set was originally indexed, integrated, and scaled using XIA2 package[28]. All structures were solved by molecular replacement method using the program PHASER[29] and the structure of WT-CALB (PDB code 1TCA)[30] as a search model. Rounds of automated refinement were performed with PHENIX[31] and the models were extended and rebuilt manually with COOT[32]. Given the fact that R_{free}/R_{factor} of QW10 was a bit high (0.2687/0.2297), we tried structural refinement under different resolution cut-off and got the lowest R_{free}/R_{factor} (0.2560/0.2160) at 2.1 Å. When we were preparing the revised manuscript, we re-processed the QW10 data-set by using XDS package[33] and got the best R_{free}/R_{factor} (0.2383/0.2135). The statistics for data collection and crystallographic refinement are summarized in Supplementary Table 4. All structural figures were prepared using Pymol (http://www.pymol.org/)[34].

Computational Methods.

Substrate *p*-nitrophenyl benzoate (**1**) was docked to the open conformation of the crystal structure of WT CALB (PDB: 5A71)[35] and relevant variants. All systems were subsequently submitted to molecular dynamics (MD) simulations. Representative conformations of the WT and variant were retrieved from cluster analysis of the MD simulations for the subsequent reaction mechanisms study using QM cluster modelling and QM/MM calculations.

Parameterization of the substrate. The parameters for the *p*-nitrophenyl benzoate were developed using the general Amber force field (GAFF)[36] within Antechamber. The charges were calculated according to the Merz–Singh–Kollman scheme[37-38] using Gaussian 09 package[39] at HF/6-31G* level. The restrained electrostatic potential (RESP)

method[40] was used for charge fitting procedure to calculate the point charges (Supplementary Dataset 1) that were subsequently used in the docking studies and MD simulations.

Molecular docking. The protonation states of the titratable residues of the WT CALB were assigned based upon previous literature[35]. The Swiss PDB Viewer was used for adding missing atoms and selecting an appropriate sidechain orientation from the rotamer library of amino acids [41]. The apo structures of the WT, QW4, and QW10 were subjected to MD simulations prior to the docking of substrate. Molecular docking was performed using the AutoDock 4.2 suite with the Lamarckian genetic algorithm (LGA) and the standard free energy scoring function [42]. For the WT CALB grid box was centred on the hydroxyl group of Ser105 side chain while for the CALB variants it was centred on the thiol group of Cys105. A total of 300 LGA runs were carried out for each ligand:protein complex. The population was 300, the maximum number of generations was 27,000 and the maximum number of energy evaluations was 2,500,000.

MD simulations. The productive MD simulations were performed using GPU version of PMEMD [43] engine integrated with Amber 16[44]. The FF14SB[45] force field was employed in all the simulations and Leap module was used to add the missing hydrogen atoms and counter ions for neutralization of the protein system. The systems were immersed into a truncated octahedral box with TIP3P[46] water molecules with the boundary of protein system being 10 Å away from box edge. The periodic boundary conditions were employed in all the simulations. Long-range electrostatic interactions have been calculated using the particle mesh Ewald[47] (PME) method with a cut-off of 8 Å for the direct space Coulomb and vdW forces.

The solute molecules were restrained using potential of 10 kcal mol^{-1} $Å^2$ and solvent and ions were subjected to energy minimization using steepest descent (5,000 steps) followed by conjugate gradient (5,000 steps) method. The entire system was then minimized with steepest descent (5,000 steps) followed by conjugate gradient (5,000 steps) method. The system was then subjected to controlled heating from 0 to 300K for 400 ps at constant volume using Langevin thermostat with a collision frequency of 1 ps^{-1} using a canonical ensemble[48]. During the heating process the solute molecules were restrained using a harmonic potential of 10 kcal mol^{-1} $Å^2$. This was followed by equilibration of the entire system at 300K for 1 ns in an NPT ensemble. A Berendsen barostat[48] was used to maintain the pressure at 1 bar and The SHAKE[49] algorithm was used to constrain bonds involving hydrogen. A time step of 2 fs and the SPFP precision model were used for all MD runs. A production MD run for continuous 100 ns was performed in a NPT ensemble with a target pressure of 1 bar and a pressure coupling constant of 2 ps. The equilibrated MD structures of WT, QW2, QW4 and QW10 variants are shown in Supplementary Datasets 3-6.

QM/MM calculations. The snapshots for the QM/MM calculations were obtained from the open conformation of the CALB enzyme and its mutants using cluster analysis of the 100ns MD trajectory using cpptraj[50]. The representative snapshots from the most populated cluster were selected.

These snapshots from MD simulations were subjected to energy minimization by 5,000-step steepest descend and 5,000-step conjugate gradient algorithms. The minimized snapshots of the WT CALB and the mutant were prepared using the Schlegel's toolkit TAO[51] for ONIOM [52-54] calculation in Gaussian09 package[39]. The enzyme-substrate complex together with the water shell of 8Å surrounding the enzyme was included in the QM/MM calculations. The residues which are within 10Å of the active site (His224, Asp187, Ser105/Cys105, Gly39, Thr40 and substrate) including water molecules were allowed to move freely during geometry optimization and the rest of the system was frozen during

geometry optimization. The total charge of the QM region was -1. The link atoms approach was used to saturate the dangling bond in the QM/MM calculation. All the calculations were run using DFT with B3LYP[55-57]/ωB97X-D[58] functional and 6-31+G (d,p) basis set. The optimized structures were taken as a starting points to perform potential energy scan (PES) along the chosen reaction coordinates in step size of 0.05 – 0.1 Å. Minima and the transition states (TS) obtained from the PES were fully optimized and were validated by frequency calculations. The TS were characterized by presence of one imaginary frequency. Single point correction were performed on the stationary points using B3LYP/6-311G++(2d,2p) basis set.

QM cluster model. The QM region was the same as used in the above QM/MM calculations. The QM calculations were performed using the Gaussian09 package. The Cβ atoms of the QM region were constrained to their X-ray structure position. The stationary points obtained from the potential energy surface scans were fully optimized and were validated by frequency calculations. All the calculations were run using DFT with B3LYP functional and 6-31+G (d,p) basis set. A conductor-like polarizable continuum model (CPCM) with ε=4.3, diethyl ether as solvent was used in the QM calculations to mimic the hydrophobic active site in the protein[59].

Supplementary References

1. Neet, K. E., Koshland, D. E., Jr. The conversion of serine at the active site of subtilisin to cysteine: A "chemical mutation". *Proc. Natl. Acad. Sci. USA* **56**(5), 1606–1611 (1966).
2. Polgar, L., Bender, M. L. A new enzyme containing a synthetically formed active site. Thiol-subtilisin. *J. Am. Chem. Soc.* **88**(13), 3153–3154 (1966).
3. Higaki, J. N., Evnin, L. B., Craik, C. S. Introduction of a cysteine protease active site into trypsin. *Biochemistry* **28**, 9256 9263 (1989).
4. Marjorie, E. W., Jeffrey, N. H., Charles S. C., Robert, J. F. Crystal structure of rat trypsin-S195C at −150 °C: Analysis of low activity of recombinant and semisynthetic thiol proteases, *J. Mol. Biol.* **219**(3), 511-523 (1991).
5. Slade, A., Horrocks, A. J., Lindsay, C. D., Dunbar, B., Virden, R. Site-directed chemical conversion of serine to cysteine in penicillin acylase from *Escherichia coli* ATCC 11105: Effect on conformation and catalytic activity. *Eur. J. Biochem.* **197**, 75-80 (1991).
6. Sigal, I. S., Harwood, B. G., Arentzen, R. Thiol-ß-lactamase: Replacement of the active-site serine of RTEM ß-lactamase by a cysteine residue. *Proc. Natl. Acad. Sci. USA* **79**, 7157-7160 (1982).
7. Li, J., Szittner, R., Derewenda, Z. S., Meighen, E. A. Conversion of serine-114 to cysteine-114 and the role of the active site nucleophile in acyl transfer by myristoyl-ACP thioesterase from vibrio harveyi. *Biochemistry* **35**, 9967-9973 (1996).
8. Chang, S. H., James, H. S. Site-directed mutagenesis of the proposed catalytic amino acids of the sindbis virus capsid protein autoprotease. *J. virol.* 3069-3073 (1990).
9. Pazirandeh, M., Chirala, S. S., Wakil, S. J. Site-directed mutagenesis studies on the recombinant thioesterase domain of chicken fatty acid synthase expressed in Escherichia coli. *J. Biol. Chem.* **266**, 20946-20952 (1991).
10. Clark, P. I., Lowe, G. Conversion of the active-site cysteine residue of papain into a dehydro-serine, a serine and a glycine residue. *Eur. J. Biochem.* **84**, 293-299 (1978).
11. Sárkány, Z., Polgár, L. The unusual catalytic triad of poliovirus protease 3C. *Biochemistry* **42**, 516-522 (2003).
12. FurgersonIhnken, L. A., Chatterjee, C., van der Donk, W. A. In vitro reconstitution and substrate specificity of a lantibiotic protease. *Biochemistry* **47**, 7352-7363 (2008).
13. Gosert, R., Dollenmaier, G., Weitz, M. Identification of active-site residues in protease 3C of hepatitis a virus by site-directed mutagenesis. *J. virology* 3062-3068 (1997).
14. Shafee, T., Gatti-Lafranconi, P., Minter, R., Hollfelder, F. Handicap-Recover evolution leads to a chemically versatile, nucleophile-permissive protease. *Chem. Bio. Chem.* **16**, 1866 -1869 (2015).
15. Mondale, M. S., Ruiz, A., Bok, D., Rando, R. R. Lecithin retinol acyltransferase contains cysteine residues essential for catalysis. *Biochemistry* **39**, 5215-5220 (2000).
16. Nelson, D., Schuch, R., Chahales, P., Zhu, S., Fischetti, V. A. Ply C: Amultimeric bacteriophage lysin. *Proc. Natl. Acad. Sci. USA* **103**, 10765-10770 (2006).
17. Tjeenk, M. L., et al. Inactivation of Staphylococcus hyicus lipase by hexadecylsulfonyl fluoride: evidence for an active site serine, *Prot. Eng.* **7**, 579 583 (1994).
18. Wong, H., et al. Lipoprotein lipase domain function, *J. Biol. Chem.* **269**, 10319 10323 (1994).
19. Sharp, J. D., et al. Serine 228 is essential for catalytic activitieso f 85-kDa cytosolic phospholipase. *J. Biol. Chem.* **269**, 23250 23254 (1994).
20. Brok, R. G. P. M., Belandia, I.U., Dekker, N., Tommassen, J. Verheij, H. M. Escherichia coli outer membrane phospholipase a: role of two serines in enzymatic activity. *Biochemistry* **35**, 7787-7793 (1996).
21. Solovskii, M. V., Nazarova, I. V., Zubko, N. V., Panarin, F. Synthesis of p-nitrophenyl esters of unsaturated phenoxyacetic acids. *Russ Chem Bull*. **32**, 624-626 (1983).
22. Huggins, C. & Lapides, J. Chromogenic substrates. IV. Acyl esters of p-nitrophenol as substrates for the colorimetric determination of esterase. *J. Biol. Chem.* **170**, 467-482 (1947).
23. Chen, C., Dai, W.C., Chang, H.G. A Facile and efficient Zinc-promoted allylation of carbonyl compounds. *Organic Prep. Proced.* **34**, 507-511 (2002).
24. Vallin, M., Syren, P., Hult, K. Mutant lipase-catalyzed kinetic resolution of bulky phenyl alkyl sec-alcohols: a thermodynamic analysis of enantioselectivity. *Chembiochem* **11**, 411-416 (2010).
25. Hogrefe, H.H., Cline, J., Youngblood, G.L., Allen, R.M. Creating randomized amino acid libraries with the QuikChange® multi site-directed mutagenesis kit. *BioTechniques* **33**, 1158–1165 (2002).

26. Sanchis, J., et al. Improved PCR method for the creation of saturation mutagenesis libraries in directed evolution: application to difficult-to-amplify templates. *Appl. Microbiol. Biotechnol.* **81**,387–397 (2008).
27. Otwinowski, Z., Minor, W. Processing of X-ray diffraction data collected in oscillation mode. *Method. Enzymol.* **276**, 307-326 (1997).
28. Graeme, W. XIA2: an expert system for macromolecular crystallography data reduction. *J. Appl. Crystallogr.* **43**, 186-190 (2010).
29. McCoy, A. J., Grosse-Kunstleve, R. W., Adams, P. D., Winn, M. D., Storoni, L.C., Read, R. J. Phaser crystallographic software. *J. Appl. Crystallogr.* **40**, 658-674 (2007).
30. Uppenberg, J., Hansen, M.T., Patkar, S., Jones, T.A. The sequence, crystal structure determination and refinement of two crystal forms of lipase B from Candida antarctica. *Structure* **2**, 293–308 (1994).
31. Adams, P. D., et al. PHENIX: a comprehensive Python-based system for macromolecular structure solution. *Acta Crystallogr. D. Biol. Crystallogr.* **66**, 213-221 (2010).
32. Emsley, P., Lohkamp, B., Scott, W. G., Cowtan, K. Features and development of Coot. *Acta Crystallogr. D. Biol. Crystallogr.* **66**, 486-501 (2010).
33. Kabsch, W. Integration, scaling, space-group assignment and post-refinement. *Acta Crystallogr D Biol Crystallogr.* **66**, 133-144 (2010).
34. DeLano, W. L. The PyMOL Molecular Graphics System; DeLano Scientific: San Carlos, CA, USA (2002) .
35. Stauch, B., Fisher, S. J. & Cianci, M. Open and closed states of Candida antarctica lipase B: protonation and the mechanism of interfacial activation. *J. Lipid Res.* **56**, 2348–2358 (2015).
36. Wang, J., Wolf, R. M., Caldwell, J. W., Kollman, P. A. & Case, D. A. Development and testing of a general amber force field. *J. Comput. Chem.* **25**, 1157–1174 (2004).
37. Besler, B. H., Merz, K. M. & Kollman, P. A. Atomic charges derived from semiempirical methods. *J. Comput. Chem*. **11**, 431–439 (1990).
38. Singh, U. C. & Kollman, P. A. An approach to computing electrostatic charges for molecules. *J. Comput. Chem.* **5**, 129–145 (1984).
39. Frisch, M.J., et al. Gaussian 09, revision D. 01. Gaussian. Inc.: Wallingford, CT (2009).
40. Bayly, C. I., Cieplak, P., Cornell, W. & Kollman, P. A. A well-behaved electrostatic potential based method using charge restraints for deriving atomic charges: the RESP model. *J. Phys. Chem.* **97**, 10269–10280 (1993).
41. Guex, N. & Peitsch, M. C. SWISS-MODEL and the Swiss-Pdb Viewer: an environment for comparative protein modeling. *Electrophoresis* **18**, 2714–2723 (1997).
42. Morris, G. M., et al. AutoDock4 and AutoDockTools4: Automated docking with selective receptor flexibility. *J. Comput. Chem.* **30**, 2785–2791 (2009).
43. Salomon-Ferrer, R., Götz, A. W., Poole, D., Le Grand, S. & Walker, R. C. Routine microsecond molecular dynamics simulations with AMBER on GPUs. 2. Explicit solvent Particle Mesh Ewald. *J. Chem. Theory Comput.* **9**, 3878–3888 (2013).
44. Case, D.A., et al. AMBER 2017, University of California, San Francisco (2017).
45. Maier, J. A., Martinez, C., Kasavajhala, K., Wickstrom, L., Hauser, K.E., Simmerling, C. ff14SB: Improving the accuracy of protein side chain and backbone parameters from ff99SB. *J. Chem. Theory Comput.* **11**, 3696–3713 (2015).
46. Jorgensen, W. L., Chandrasekhar, J., Madura, J. D., Impey, R. W. & Klein, M. L. Comparison of simple potential functions for simulating liquid water. *J. Chem. Phys.* **79**, 926–935 (1983).
47. Darden, T., York, D. & Pedersen, L. Particle mesh Ewald: An N·log(N) method for Ewald sums in large systems. *J. Chem. Phys.* **98**, 10089–10092 (1993).
48. Berendsen, H. J. C., Postma, J. P. M., van Gunsteren, W. F., DiNola, A. & Haak, J. R. Molecular dynamics with coupling to an external bath. *J. Chem. Phys.* **81**, 3684–3690 (1984).
49. Ryckaert, J. P., Ciccotti, G. & Berendsen, H. J. C. Numerical integration of the cartesian equations of motion of a system with constraints: molecular dynamics of n-alkanes. *J. Comput. Phys.* **23**, 327–341 (1977).

50. Roe, D. R. & Cheatham, T. E. PTRAJ and CPPTRAJ: Software for processing and analysis of molecular dynamics trajectory data. *J. Chem. Theory Comput*. **9**, 3084–3095 (2013).
51. Tao, P. & Schlegel, H. B. A toolkit to assist ONIOM calculations. *J. Comput. Chem.* **31**, 2363–2369 (2010).
52. Maseras, F. & Morokuma, K. IMOMM: A new integrated ab initio + molecular mechanics geometry optimization scheme of equilibrium structures and transition states. *J. Comput. Chem.* **16**, 1170–1179 (1995).
53. Svensson, M. et al. ONIOM: A multilayered integrated MO + MM method for geometry optimizations and single point energy predictions. A test for Diels−Alder reactions and Pt(P(t-Bu)3)2 + H2 oxidative addition. *J. Phys. Chem.* **100**, 19357–19363 (1996).
54. Dapprich, S., Komáromi, I., Byun, K. S., Morokuma, K. & Frisch, M. J. A new ONIOM implementation in Gaussian98. Part I. The calculation of energies, gradients, vibrational frequencies and electric field derivatives. *J. Mol. Struct. THEOCHEM* **461–462**, 1–21 (1999).
55. Becke, A. D. Density-functional exchange-energy approximation with correct asymptotic behavior. *Phys. Rev.* A **38**, 3098–3100 (1988).
56. Becke, A. D. Density-functional thermochemistry. III. The role of exact exchange. *J. Chem. Phys.* **98**, 5648-5652 (1993).
57. Lee, C., Yang, W., Parr, R.G. Development of the Colle-Salvetti correlation-energy formula into a functional of the electron density. *Phys. Rev. B Condens. Matter* **37**, 785–789 (1988).
58. Chai, J.D., Head-Gordon, M. Long-range corrected hybrid density functionals with damped atom-atom dispersion corrections. *Phys. Chem. Chem. Phys.* **10**, 6615–6620 (2008).
59. Barone, V. & Cossi, M. Quantum calculation of molecular energies and energy gradients in solution by a conductor solvent model. *J. Phys. Chem. A* **102**, 1995–2001 (1998).
