# Supplementary material for: Artificial cysteine-lipases with high activity and altered catalytic mechanism created by laboratory evolution
Source: Nat Commun. 2019 Jul 19;10:3198. doi: 10.1038/s41467-019-11155-3 (PMC6642262; doi:10.1038/s41467-019-11155-3)
Supplement: Supplementary file 3 — Description of Additional Supplementary Files [file 41467_2019_11155_MOESM3_ESM.pdf]

## Description of Additional Supplementary Files

File Name: Supplementary Dataset 1
Description: The force field parameters for p-nitrophenyl benzoate.

File Name: Supplementary Dataset 2
Description: The coordinates of QM/MM stationary points of QW10 and QW4 mutants.

File Name: Supplementary Dataset 3
Description: the PDB file of the docked structures and the equilibrated MD structures of WT.

File Name: Supplementary Dataset 4
Description: the PDB file of the docked structures and the equilibrated MD structures of QW2.

File Name: Supplementary Dataset 5
Description: the PDB file of the docked structures and the equilibrated MD structures of QW10.

File Name: Supplementary Dataset 6
Description: the PDB file of the docked structures and the equilibrated MD structures of QW4.
